# A Split-Ubiquitin Yeast Two-Hybrid Screen to Examine the Substrate Specificity of atToc159 and atToc132, Two Arabidopsis Chloroplast Preprotein Import Receptors

**DOI:** 10.1371/journal.pone.0095026

**Published:** 2014-04-15

**Authors:** Siddhartha Dutta, Howard J. Teresinski, Matthew D. Smith

**Affiliations:** Department of Biology, Wilfrid Laurier University, Waterloo, Ontario, Canada; University of California - Davis, United States of America

## Abstract

Post-translational import of nucleus-encoded chloroplast pre-proteins is critical for chloroplast biogenesis, and the Toc159 family of proteins serve as receptors for the process. Toc159 shares with other members of the family (e.g. Toc132), homologous GTPase (G−) and Membrane (M−) domains, but a highly dissimilar N-terminal acidic (A−) domain. Although there is good evidence that atToc159 and atToc132 from Arabidopsis mediate the initial sorting step, preferentially recognizing photosynthetic and non-photosynthetic preproteins, respectively, relatively few chloroplast preproteins have been assigned as substrates for particular members of the Toc159 family, which has limited the proof for the hypothesis. The current study expands the number of known preprotein substrates for members of the Arabidopsis Toc159 receptor family using a split-ubiquitin membrane-based yeast two-hybrid system using the atToc159 G-domain (Toc159G), atToc132 G-domain (Toc132G) and atToc132 A- plus G-domains (Toc132AG) as baits. cDNA library screening with all three baits followed by pairwise interaction assays involving the 81 chloroplast preproteins identified show that although G-domains of the Toc159 family are sufficient for preprotein recognition, they alone do not confer specificity for preprotein subclasses. The presence of the A-domain fused to atToc132G (Toc132AG) not only positively influences its specificity for non-photosynthetic preproteins, but also negatively regulates the ability of this receptor to interact with a subset of photosynthetic preproteins. Our study not only substantiates the fact that atToc132 can serve as a receptor by directly binding to chloroplast preproteins but also proposes the existence of subsets of preproteins with different but overlapping affinities for more than one member of the Toc159 receptor family.

## Introduction

Chloroplasts are specialized plastids, which are not only the site of photosynthesis, but also play crucial roles in other essential processes such as lipid, phytohormone and amino acid biosynthesis [1], [2], nitrogen and sulphur metabolism [3], and chloroplast-to-nucleus retrograde signaling [4]. Because of their semi-autonomous nature [5], the biogenesis of chloroplasts requires a well coordinated and tightly regulated interaction between the nuclear and chloroplast genomes [4]. The vast majority of chloroplast proteins are encoded in the nucleus [6], [7]. To maintain the optimal stoichiometry of the chloroplast proteome, the majority of nucleus-encoded chloroplast precursor proteins are transported into the organelle through the hetero-oligomeric Toc-Tic (Translocon at the Outer/Inner envelope membrane of Chloroplast) protein complexes that are localized at the chloroplast envelope [8], [9], [10], [11]. Chloroplast-destined preproteins carry a distinguishing N-terminal transit peptide (TP) of 10–150 amino acids that is necessary and sufficient for targeting preproteins to the chloroplast stroma [12], [13], [14]. TPs are recognized by one or more members of the two families of Toc GTPase receptors, Toc159 and Toc34, which are core components of the Toc complex [15], [16], [17], [18], [19], [20]. Extensive studies on the Toc159 and Toc34 families of GTPase receptors and the import of photosynthetic and constitutive non-photosynthetic pre-proteins have given rise to the hypothesis that there are multiple import pathways in Arabidopsis, which are responsible for maintaining chloroplast homeostasis by supporting proportionate import of variably expressed nucleus-encoded chloroplast proteins at different stages of chloroplast biogenesis [21], [22], [23], [24].

Genetic and biochemical studies in *Arabidopsis thaliana* have revealed the presence of multiple homologues of the two GTPase receptor families, which contribute to the formation of structurally distinct Toc complexes, and are thought to be functionally specific for distinct sets of preproteins [8], [23]. The Toc159 family of receptors is represented by four homologues in Arabidopsis, namely, atToc159, atToc132, atToc120 and atToc90 [25], [26], [27], [28]. atToc159 is the most abundant homologue of the family in green tissues [25], [28]. It shares a distinct 3-domain structure with the other members of the family, consisting of an intrinsically unstructured acidic (A−) domain at its N-terminus, a central GTP-binding (G−) domain, and a C-terminal membrane-binding (M−) domain [29], [30]. All of the Toc159 homologues contain similar G- and M- domains but highly dissimilar N-terminal A-domains [29], which is totally lacking in the case of atToc90 [26]. Mutant analysis and binding assays have revealed two primary forms of the Toc complex: one comprised of atToc159/90-atToc33-atToc75, thought to be primarily responsible for the import of photosynthetic preproteins, and the other consisting of atToc132/120-atToc34-atToc75, thought to be primarily responsible for recognizing and importing constitutive preproteins that are not necessarily involved in photosynthesis [8], [19], [29], [31]. Analysis of an atToc159 knock-out mutant (*ppi2*) places this Toc component high in the functional hierarchy, owing to defects in chloroplast biogenesis, accumulation of photosynthetic preproteins and an albino, seedling lethal phenotype in plants lacking this component [25]. None of the mutants for alternate homologues of atToc159 (i.e. atToc132/120/90) yielded any severe phenotypes [26], [28], [29]. However, double mutants lacking both atToc132 and atToc120 *(toc120-1/toc132-1*) exhibit variable, and in some cases severe, defects in phenotype as observed in separate studies, suggesting their critical role in import and functional redundancy [28], [29]. More recently, atToc90 was shown to partially complement the *ppi2* phenotype, suggesting an overlapping role between atToc90 and atToc159 [32].

The Toc34 family is represented by two functionally overlapping homologues in Arabidopsis, atToc34 and atToc33 [8], [33]. atToc33 mutants (*ppi1*) display a mild phenotypic defect at early stages of development with accumulation of photosynthetic preproteins, which suggests a function in maintaining the photosynthetic proteome coordinately with atToc159 [8], [33], [34]. Collectively, the available data has led to the working hypothesis that Toc complexes consisting of atToc159 and atToc33 are the major contributors of photosynthetic preprotein recognition and import, whereas atToc132/120 in conjunction with atToc34 are hypothesized to function together in the recognition and import of non-photosynthetic proteins [8], [10], [31].

Although progress has been made in our understanding of TP recognition by the Toc complex, there is still much to be determined owing to the large number of preproteins targeted to chloroplasts (∼2100) and the highly diverse transit peptidome [13], [14], [35], [36]. Chemical crosslinking studies of preproteins during different stages of import into chloroplasts provided early evidence that the G-domain of Toc159 is the primary binding site for transit peptides [16], [17]. This was reinforced by more recent direct preprotein pull down assays using the G-domain as bait [19]. A role for the A-domain in functional differentiation among Toc159 homologues has long been hypothesized due to its variability between homologues [25], [29], and the demonstration that this domain is intrinsically unstructured has bolstered the hypothesis that it may play a role in conferring specificity to the homologues for distinct sets of preproteins [30]. *In vitro* and *in vivo* assessments of the function of the Toc159 receptor family through deletion mutations and swapping of the variable A-domains suggest that this domain may be an important contributor to the differential import of photosynthetic and non-photosynthetic preproteins [29], [37]. A-domain truncation mutants of atToc159 and atToc132, denoted atToc159GM and atToc132GM, fail to exhibit selectivity towards photosynthetic and non-photosynthetic TPs [37].

The nature of the interaction between a TP and receptor is largely directed by the TP sequence and its physico-chemical properties (e.g. charge, structure) [13], [14], [38]. A series of studies have revealed a few common characteristics of TPs, such as deficiency of acidic amino acids, high abundance of hydroxylated amino acids and the presence of short, defined motifs [38], [39], [40], [41]. Bioinformatics studies on 208 chloroplast preproteins were used to identify a common specific motif of TPs, and resulted in the identification of seven different subgroups, which are highly dissimilar in their properties [35], [42]. It is of note that both TPs and the A-domain of the Toc159 receptor family have been shown to be intrinsically disordered proteins (IDPs), which are often involved in protein-protein interactions, and are thought to allow for highly specific, yet low affinity interactions with a large number of binding partners [30], [38], [43]. In spite of several proposed hypotheses [30], [35], [38], a conserved mechanism for the interaction between TPs and their receptors is still not agreed upon. Thus with ∼2100 different nuclear-encoded precursors that are targeted to Arabidopsis chloroplasts and a much smaller number of pathways to accommodate their import, it is imperative to extend our understanding of how such a large number of preproteins/TPs are recognized by such a small number of receptors. The current hypothesis regarding import of different subsets of preproteins by atToc159-atToc33 and atToc132/120-atToc34 containing Toc complexes is based on a very small number of preprotein substrates. Expanding the number of preproteins known to interact with the different members of the Toc159 family of receptors would be a valuable way of testing the hypothesis. Therefore, in the present study, we aimed to increase the number of known preprotein substrates for atToc159 and atToc132 in Arabidopsis, and thus shed more light on the inherent preprotein binding properties of these receptors.

Several methods, including *in vitro* protein-protein interaction studies, microarray analysis and *in vivo* approaches have been extensively employed to investigate protein-protein interaction properties of many different proteins [44], [45], [46]. The identification and characterization of most of the known components of the chloroplast protein import machinery, as well as their interaction with TPs, are based on extensive usage of protein fractionation, BN-PAGE, *in vitro* and *in vivo* pull-down assays and biophysical techniques [19], [37], [47], [48], [49], [50], [51]. An advantage of using these approaches was their ability to reveal physiologically relevant interactions governing the recognition and transport of preprotein substrates through the translocon complexes [19], [37], [47], [50], [51], [52]. On the other hand, a shortcoming of these studies has been the number of different preproteins that have been considered, due to limitations of the methodologies. Among the various alternative approaches that are available to study protein-protein interactions, the yeast two-hybrid system has been used successfully to study the chloroplast protein import apparatus [50], [53], [54]. This approach hasn’t been used extensively, however, because in conventional yeast-two hybrid systems, candidate proteins must be in a soluble form and protein interactions occur in the nucleus of the yeast cells [55], [56], [57]. In order to increase the number of assigned preprotein substrates for members of the Toc159 receptor family, we used a split-ubiquitin membrane-based yeast two-hybrid screening system [58]. Apart from being able to carry out a large-scale study of preprotein interaction partners in live cells, this system also provided us the flexibility of studying both soluble and membrane-bound proteins [59], [60], [61]. In addition, because the interaction between bait and prey proteins occurs in the cytoplasm or at a membrane, rather than in the nucleus, it provides a more physiological environment for the interaction than a conventional nucleus-based yeast two-hybrid system for the Toc159/132 receptors and their putative interactors [62]. The G-domain of Toc159 is known to interact with the TPs of precursor proteins [19]. Therefore, we carried out our initial screening utilizing the G-domains of atToc159 and atToc132 as baits for screening an Arabidopsis cDNA library of potential prey proteins. We wished to extend our analysis to include atToc159 and atToc132 bait proteins that included their A-domains in addition to the G-domains. Unfortunately, we were unable to produce a functional atToc159 bait protein that included its A-domain in yeast cells; therefore, this part of the analysis was limited to an examination of the binding partners of an atToc132 bait protein consisting of it G- plus A-domain. To further investigate the role of the A-domain in determining the specificity of receptors for certain preproteins, we analyzed the binding of a subset of TP fusion proteins against the atToc159 receptor family using *in vitro* pull down assays. Apart from adding to the list of known preprotein substrates for members of the Toc159 receptor family, our results point toward a less stringent preprotein specificity for the Toc159 receptors than has been hypothesized. Our investigation does reaffirm the previously proposed regulatory role for the A-domain in preprotein selectivity; but, it also cautions us about choosing specific preproteins as candidates for interaction studies, owing to the high variability observed for the affinity of different photosynthetic and non-photosynthetic preproteins for the different members of the Toc159 family of receptors. The study not only contributes to our knowledge regarding preprotein recognition by the Toc159/132 receptors at the chloroplast surface but also opens the door to future *in vitro* and *in vivo* investigations aimed at understanding the detailed molecular determinants of preprotein recognition and binding, and how they relate to the overall mechanism of preprotein import into chloroplasts.

## Materials and Methods

### Plant Materials and Growth Conditions

Seeds of *Arabidopsis thaliana* ecotype Columbia were surface sterilized in 95% ethanol for 5 min, followed by 20 min in 30% bleach containing 0.02% (v/v) Triton X-100, washed five times in sterile water and sown on 150 mm×15 mm plates containing 0.5× Murashige and Skoog media and 0.8% agar. Seeds were stratified at 4°C for 48 h. Plants were grown for 10 days at 22°C under long-day conditions (16 hours light/8 hours dark) in a controlled growth chamber (Enconair, Bigfoot Series).

### Yeast Strains and Manipulations

All work with yeast was done using *Saccharomyces cerevisiae* strain NMY51:MATahis3Δ200 trp1-901 leu2-3,112 ade2 LYS2::(lexAop)_4_-HIS3 ura3::(lexAop)_8_-lacZ ade2::(lexAop)_8_-ADE2 GAL4 [63]. Yeast cells were grown using standard microbial techniques and media [63], [64]. Media designations are as follows: YPAD is Yeast Extract - Peptone - Dextrose plus Adenine medium; SD is Synthetic Defined dropout (SD-drop-out) medium. Minimal dropout media are designated by the constituent that is omitted (e.g. -leu –trp –his –ade medium media lacks leucine, tryptophan, histidine and adenine). Recombinant plasmid DNA constructs were introduced into NMY51 by LiOAc-mediated transformation as described [65].

### Arabidopsis cDNA Library Construction

For constructing cDNA libraries, mRNA was isolated from 10-day old Arabidopsis seedlings using the PolyA-Tract System 1000 (Promega) according to the manufacturer’s instructions. The quality and quantity of the Poly(A) RNA were determined using agarose gel electrophoresis and UV absorption spectrophotometry. cDNA was synthesized using the Dualsystems Biotech EasyClone cDNA synthesis kit, according to the manufacturer’s instructions. In short, poly(A)+ mRNA derived from 10-day old Arabidopsis seedlings was used to direct the synthesis of first-strand cDNA by reverse transcriptase either with an SfiI-oligo-dT primer 5′- AAGCAGTGGTATCAACGCAGAGTGGCCGAGGCGGCC(T)_20_VN-3′ or SfiI-random primer 5′-AAGCAGTGGTATCAACGCAGAGTGGCCGAGGCGGCC(N)_6_-3′ for the oligo-dT primed and random-primed cDNA library construction, respectively. A PlugOligo-3M adapter 5′- AAGCAGTGGTATCAACGCAGAGTGGCCATTACGGCCGGGGG-P-3′ was incorporated to the first-strand cDNA per the manufacturer’s instructions (Dualsystems Biotech). Following second-strand synthesis, the cDNAs were digested with SfiI, and consequently size-selected using CHROMASPIN™-400 columns (Clontech) following the manufacturer’s instructions. Fractions containing cDNAs ≥500 kb in length were pooled and subcloned into the yeast library plasmid pR3-N and pR3-C for oligo-dT and random-primed cDNA libraries, respectively (Dualsystem Biotech). The average insert size was evaluated by purifying plasmid DNA from 48 random clones and digesting with SfiI. Each library had an average complexity of 2×10^6^ transformants and an average insert size of 1.2 kb. Finally, a total of approximately 2×10^6^ independent clones were collected, combined and grown in LB, from which plasmid maxi-preparations were performed (Qiagen).

### Construction of Plasmid Vectors

#### Bait vectors for expression in yeast

To screen for potential protein interaction partners, a LexA-VP16 DNA binding domain-based vector (pBT-3 STE) supplied by the manufacturer (Dualsystems Biotech) was used as a bait vector. DNA fragments corresponding to atToc159G (amino acids 228–1092), atToc132AG (amino acids 1–794) and atToc132G (amino acids 456–749) were amplified by PCR using pET21d-atToc159 and pET21a-atToc132, respectively, as templates [25], [29], [66]. PCR-amplified 159G, 132G and 132AG fragments were inserted into the vector using the unique SfiI restriction site of pBT-3 STE (Dualsystem Biotech), resulting in three different bait vectors encoding fusion proteins consisting of the bait proteins upstream of the C–terminal Cub-LexA-VP16 fusion partner. The inserts were confirmed to be in-frame with the C-terminal Cub-LexA-VP16 and to be free of mutations by sequencing (TCAG Sequencing Facility, Hospital for Sick Children, Toronto, ON).

#### Vector constructs for bacterial expression

The plasmid encoding pET21d-DHFR_His_ has been described previously [19]. Coding sequences for the transit peptide of LHCA4 and FNR1 were amplified from the *A. thaliana* cDNA and fused in-frame with the coding sequence of DHFR_His_ to generate pET21d-LHCA4(TP)-DHFR_His_ and pET21d-FNR1(TP)-DHFR_His._ Both of the constructs were directionally cloned using the NdeI/NcoI restriction sites.

#### Vector constructs for in vitro translation

A cDNA encoding the A- plus G-domains of atToc159 (atToc159AG, corresponding to amino acids 1–1092) was cloned in the NcoI and XhoI sites of pET21d (Novagen) to generate pET21d-atToc159AG_No-His_. A cDNA fragment corresponding to the A- and G-domains of atToc132 (atToc132AG, amino acids 1–794) was amplified by PCR using pET21a-atToc132 as the template. The insert was cloned into the pET21a (Novagen) expression vector in the forward direction using the NdeI/SacI restriction sites with a stop codon introduced at the end of the G-domain using the reverse primer to generate the pET21a-atToc132AG_No-His_ construct.

### Yeast Two-hybrid Screens

Large-scale yeast library transformation was performed as described by the DUALmembrane starter kit (Dualsystems Biotech). The yeast strain NMY51 (Dualsystem Biotech) was first transformed with one of the three different bait constructs. Titration with 3-Aminotriazole (3-AT), a competitive inhibitor of the *HIS3* gene product, for each bait was performed by co-expressing each bait with the empty pR3-N library vector (Dualsystems Biotech). The 3-AT concentration required to prevent auto-activation was determined to be 10 mM for atToc159G, 40 mM for atToc132G and 50 mM for atToc132AG. The yeast strain NMY51 containing the bait vector was grown in Synthetic Defined-Leu (SD-L) liquid medium at 30°C for 8 h with shaking. The starter cultures were used to inoculate 100 ml of fresh SD-L media and were grown again at 30°C overnight with shaking. OD_546_ of the overnight culture was measured and the amount of culture needed corresponding to 30 OD_546_ units was pelleted down and resuspended in 200 ml of 2× YPAD medium (final OD_546_ = 0.15). The resulting cultures were grown at 30°C on a shaker until OD_546_ = 0.6. The cells from each of the cultures were harvested in four 50-ml conical screw-cap centrifuge tubes, and each pellet was washed using 30 ml of sterile water before finally being resuspended in a total of 600 µl LiOAc/TE mastermix (100 mM LiOAc, 1× TE, pH 7.5) and transferred to new 50-ml conical screw-cap centrifuge tubes. 100 µl of salmon sperm DNA (20 mg/ml) and 10 µg of the selected prey library were mixed and added to each of the three bait-containing cultures. 2.5 ml of PEG/LiOAc mix (100 mM lithium acetate, 40% PEG 3350, 1× TE) was added to the cells and vortexed followed by incubation at 30°C for 45 min. Thereafter, 160 µl of DMSO was added to each tube, mixed immediately by gentle shaking followed by a 20 min heat shock at 42°C. Cells from each tube were harvested by centrifugation (700×g for 5 min), resuspended in 3 ml of 2× YPAD medium and pooled. The cells were allowed to recover at 30°C for 90 min with slow shaking (150 rpm). Afterward, cells were washed and resuspended in 4.8 ml of 0.9% NaCl. For the determination of transformation efficiency, 100 µl of 1∶100, 1∶1000 and 1∶10000 serial dilutions in 0.9% NaCl from the cell suspension were plated on SD medium lacking leucine and tryptophan (SD-LW) and incubated at 30°C. The rest of the recovered cells were plated on SD medium lacking leucine, tryptophan, histidine and adenine (SD-LWHA) supplemented with 3-AT on fifteen 150 mm plates and incubated for 6 days at 30°C. After 4 days, colonies on individual SD-LW plates were counted and the total number of transformants was calculated. To verify putative interactions between bait proteins, and prey proteins from the cDNA libraries, positive clones from the screens were re-streaked on SD-LWHA/3-AT media. For those strains growing after 5 days on SD-LWHA/3-AT media, prey plasmids were recovered as described previously [67]. The DNA was analyzed by restriction digest and also tested for bait dependency by retransformation into a yeast strain containing the respective bait plasmid or non-interaction control bait, pTSU2-APP (Dualsystems Biotech). Finally, the identity of the positive clones was determined by sequencing and using Basic Local Alignment Search Tool (BLAST) searches.

### β-Galactosidase (β-gal) Activity Assay

Quantitative β-galactosidase (β-gal) activity assays of *S. cerevisiae* extracts were carried out as previously described [68], [69], [70] using the High Throughput β-Galactosidase Assay Kit (HTX, Dualsystems Biotech) and the manufacturer’s protocol. In short, several 2-day old yeast colonies harboring each interaction pair were inoculated in 5 ml of SD-LW liquid medium and grown with shaking (250 rpm) at 30°C until the culture reached an OD_546_ of approximately 0.8. An amount of culture needed for 0.5 OD_546_ units was removed, the cells were harvested by centrifugation and the pellet resuspended in 100 µl of lysis mixture (HTX Kit, Dualsystems Biotech) containing dye solution. Samples were gently resuspended, transferred to a 96-well microplate and colour development was monitored at 615 nm using a microplate reader (BioTeK, USA). The β-Gal activity was quantified using the equation, (1000×A_615_)/(t×V×OD_546_) where t is the incubation time (min) and V is the volume of the cells used for the assay (ml). Qualitative β-gal activity assays were carried out using a colony-lift filter assay as previously described [71] and instructions from the yeast protocol handbook (Clontech).

### *In vitro* Translation and Protein Expression in E. coli

All [^35^S]methionine-labeled *in vitro* translation products ([^35^S]atToc159AG and [^35^S]atToc132AG) were generated in a coupled transcription-translation system containing rabbit reticulocyte lysate according to the manufacturer’s recommendations (Promega, Madison, WI). For bacterial overexpression, pET21d-LHCA4(TP)-DHFR_His_, pET21d-FNR1(TP)-DHFR_His_ and pET21d-DHFR_His_ were transformed into *E. coli* BL21 Codon Plus (Stratagene). Expression of LHCA4(TP)-DHFR_His_, FNR1(TP)-DHFR_His_ and DHFR_His_ was achieved by induction with 0.3 mM isopropyl β-D-thiogalactoside for 2.5 h at 37°C. The overexpressed hexahistidine-tagged proteins were purified from the insoluble inclusion body fraction of the bacterial lysate under denaturing conditions in the presence of 6 M urea using Ni^2+^-NTA chromatography (Novagen, Madison, WI) as described previously [19].

### Solid Phase Binding Assays

Direct interaction between LHCA4(TP)-DHFR_His_, FNR1(TP)-DHFR_His_ or DHFR_His_ and the AG- domains of atToc159 or atToc132 were measured using solid phase binding assays as described previously [29], [66], [72]. Varying concentrations of purified hexahistidine-tagged LHCA4(TP)-DHFR_His_, FNR1(TP)-DHFR_His_ or DHFR_His_ were diluted in 6 M urea to give a final concentration of 20 mM imidazole. The samples were immobilized on ∼8 µl of packed Ni^2+^-NTA resin at 25°C for 30 min under constant mixing. The resin was washed twice with 250 µl of binding buffer (50 mM Hepes-KOH, pH 7.5, 5 mM MgCl_2,_ 40 mM KOAc, 0.1% Triton X-100) containing 40 mM imidazole and 0.1 mM GTP. The resin was then incubated with 1 µl of [^35^S]atToc159AG or [^35^S]atToc132AG *in vitro* translation product in binding buffer containing 40 mM imidazole and 0.1 mM GTP in a final volume of 100 µl for 30 min at 25°C, under constant mixing. The resin was washed three times with 300 µl of ice cold binding buffer containing 40 mM imidazole and 0.1 mM GTP. Proteins were eluted from the resin using SDS-PAGE sample buffer containing 0.5 M imidazole and resolved by SDS-PAGE. Gels were stained with Coomassie blue to detect LHCA4(TP)-DHFR_His_, FNR1(TP)-DHFR_His_ or DHFR_His_ (data not shown), and [^35^S]atToc159AG or [^35^S]atToc132AG were detected in dried gels using a Personal Molecular Imager FX phosphorimager (Bio-Rad Laboratories Ltd).

### Immunoblot Analysis

Expression of 159G and 132G bait proteins was confirmed by extracting total protein from yeast cells as described in the DUALmembrane starter kit (Dualsystem Biotech), and proteins were detected using Western blot analysis according to standard methods [30]. Total protein extracts were resolved using SDS-PAGE, transferred onto a nitrocellulose membrane (Millipore) and blocked with 3% BSA. The blots were probed with mouse monoclonal anti-LexA antibody (Dualsystem Biotech) to detect the expression of the bait fusion proteins. Peroxidase-conjugated rabbit anti-mouse IgG (Rockland) diluted 1∶5000 was used to facilitate chemiluminescent detection. Immunoreactive bands were visualized using a Bio-Rad Fluor-S MultiImager and images were analyzed using Quantity One 1-D Analysis software v4.6 (Bio-Rad Laboratories Inc).

### Statistical Analysis

Statistical significance of differences in relative β-Gal activity, comparing two independent groups were evaluated by using the unpaired, two-tailed *t-*test. Probability levels p≤0.05 were taken as statistically significant.

## Results

To investigate the selectivity of chloroplast preprotein recognition and binding by the atToc159 family of receptors, we screened two Arabidopsis cDNA libraries using a split-ubiquitin membrane-based yeast two-hybrid system with the G-domain of atToc159 (Toc159G), the G-domain of atToc132 (Toc132G), and a construct that included both the A- and G-domains of atToc132 (Toc132AG) as the bait. In order to maximise the complexity of the preproteins being screened, both random- and oligo(dT)-primed Arabidopsis cDNA libraries suitable for split-ubiquitin membrane-based yeast two-hybrid screening were prepared.

### Generation of NMY51 Strains of S. cerevisiae Expressing Functional Toc159 Receptor Family Bait Proteins

**Figure 1:**
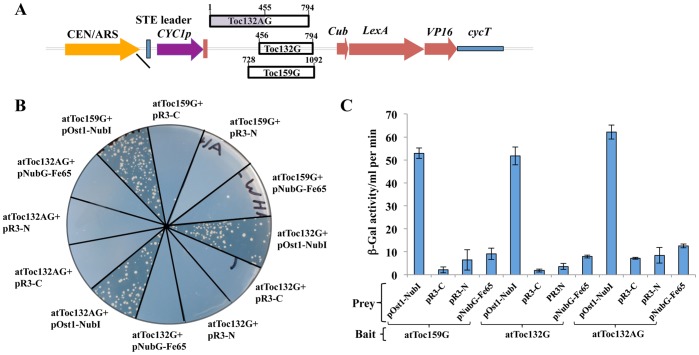
Analysis of yeast clones expressing the functional atToc159G, atToc132G and atToc132AG bait proteins. (A) Diagrammatic representation of the domain organisation of the atToc159G, atToc132G and atToc132AG bait constructs in the yeast plasmid, pBT3-STE. The bait vector provides an upstream yeast STE2 leader sequence and yeast ubiquitin Cub (34–76 aa), LexA and VP16 genes downstream. Fusion proteins produced by this cassette are expressed constitutively by the yeast CYC1 promoter and terminator. The bait protein domains were cloned directionally (using *Sfi*I) into the position indicated. The numbers refer to the amino acid sequence of atToc159 or atToc132. (B) The split-ubiquitin membrane based yeast two-hybrid analysis confirming expression of the bait proteins. atToc159G, atToc132G or atToc132AG bait was co-expressed in the *S. cerevisiae* strain NMY51 with the positive prey construct pOst1-NubI, empty prey vector, pR3-C/pR3-N or the non-interacting negative control construct pNubG-Fe65 and assayed on quadruple selective media (SD-LWHA) plates. Strains co-expressing bait protein and positive control prey exhibit growth only on SD-LWHA selective media. (C) Quantitative β-Galactosidase activity assay. Strains co-expressing respective bait and prey constructs were used in a microtitre plate-based β-galactosidase assay using 5-bromo-4-chloro-3-indolyl-β-D-galactopyranoside (X-Gal) as a substrate. The graph shows β-galactosidase activity/ml per min measured after 60 min. The values represent the mean of three independent experiments. The assay confirmed the functionality of the bait as the atToc159G, atToc132G and atToc132AG fusions interact with positive control prey but not with the empty vectors or negative control prey.

The domains of interest from the family of Arabidopsis Toc159 chloroplast preprotein receptors (Toc159G, Toc132G and Toc132AG) were cloned into the bait vector pBT3-STE. For the Toc159G bait construct, a PCR clone corresponding to amino acids 728–1092 of atToc159 was cloned. Similarly, the baits for Toc132G and Toc132AG were generated through cloning of cDNA fragments of atToc132 encoding amino acids 456–794 and 1–794, respectively. The cDNA fragments encoding the bait proteins were cloned into the pBT3-STE vector such that they were upstream and in-frame with the Cub (C–terminus of yeast ubiquitin, amino acids 34–76) and LexA-VP16 transcription factor coding sequences (Figure 1A). All the baits were preceded by a weak CYC promoter and a STE2 leader sequence corresponding to the N-terminal 13 amino acids of the *S. cerevisiae* Ste2 protein for targeting the resulting heterologous proteins to the yeast ER membrane [73]. The expression of the baits in yeast was confirmed by immunoblots in the case of atToc159G and atToc132G (Figure S1A), and using a functional assay for all three baits examined (Figure 1B).

In order for a bait to be functional in the split-ubiquitin membrane-based yeast two-hybrid system, it must be inserted in the proper orientation into the yeast ER membrane such that its interacting domains are available to interact with potential membrane-bound or cytosolic prey proteins. To ensure their functionality, the Toc159G, Toc132G and Toc132AG baits were co-expressed with positive prey control protein pOstI-NubI in yeast strain NMY51. Ost1, the gene product of OstI, is a yeast resident ER protein, and is not known to interact with any member of the Toc159 family of receptors. However, the fusion partner of Ost1, NubI, has a high affinity for Cub, meaning the yeast reporter system will be activated independently of any interaction between bait and prey proteins if a functional version of the bait is being produced. The co-expression of each of the 3 different bait proteins and the pOstI-NubI reporter prey protein resulted in the activation of reporter genes, evidenced by robust growth of yeast on the highly stringent quadruple dropout medium (SD-Leu-Trp–His-Ade; Figure 1B). As a negative control, cells were co-transformed with the Toc159G, Toc132G or Toc132AG bait plasmids and empty vectors, pR3-C/pR3-N or a non-interacting prey control, pNubG-Fe65. None of the co-transformants expressing any of the three baits and empty vectors or negative control prey grew on the quadruple dropout medium (Figure 1B). All of the NMY51 yeast cells co-expressing the bait proteins with positive or negative control prey or harboring empty prey plasmids grew robustly on the double dropout medium, SD-Leu-Trp (data not shown). The strength of interactions was confirmed by quantitative β-galactosidase activity assays (Figure 1C). The β-galactosidase activity (β-Gal activity ml^–1^ min^–1^) was highest in cells co-expressing functional baits (i.e. Toc159G, Toc132G or Toc132AG) with the positive control prey (pOsTI-NubI), indicating a strong interaction, and that the bait proteins were functional. On the contrary, β-Gal activity was very low in cells co-transformed with the bait proteins and either empty prey vectors or negative control prey constructs (Figure 1C), indicating that none of the baits had significant intrinsic activation properties.

### Screening of Arabidopsis cDNA Libraries with Toc159G, Toc132G and Toc132AG Bait Proteins

**Figure 2:**
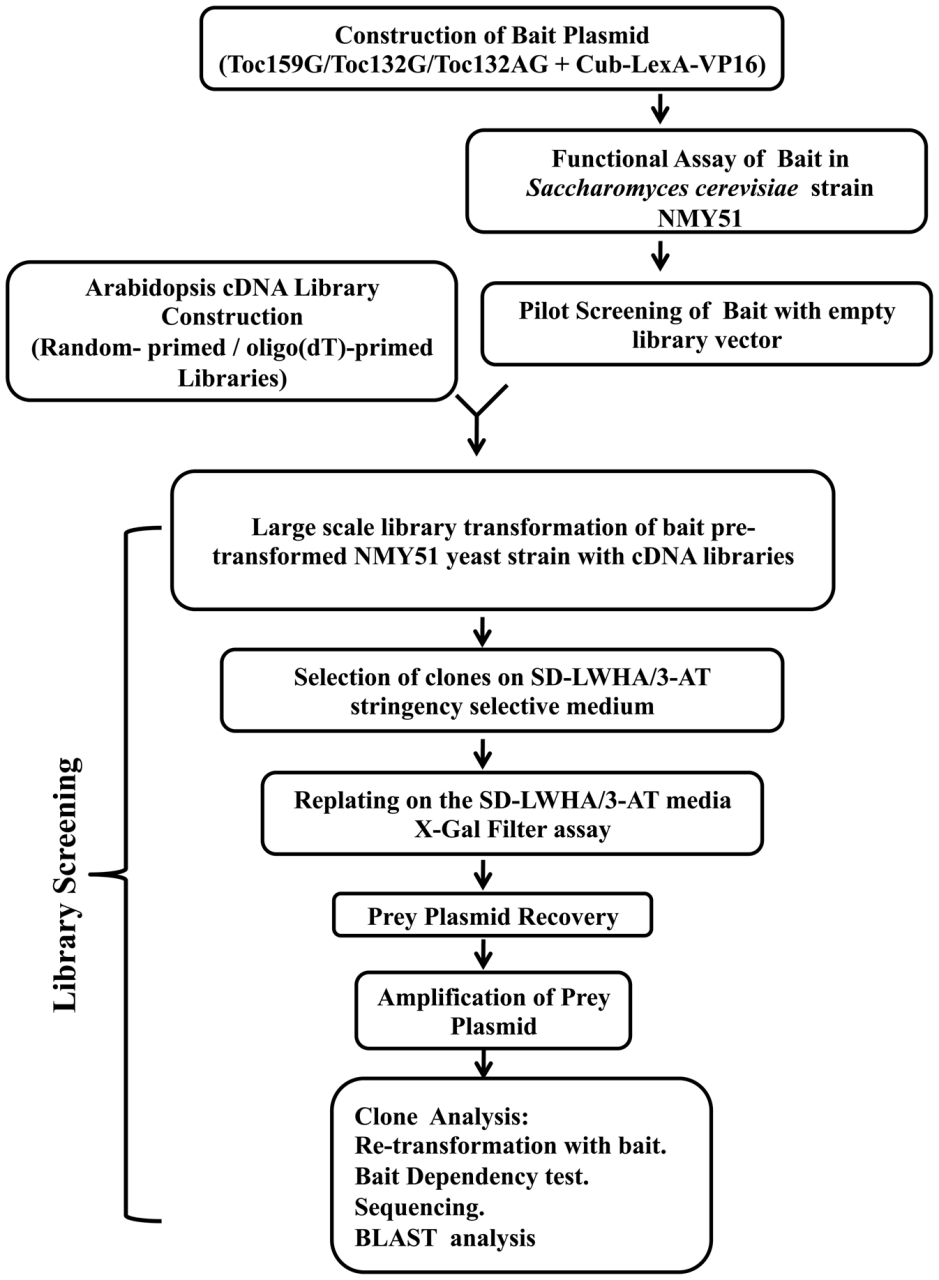
Flow chart outlining the protocol used for split-ubiquitin-based screening of an Arabidopsis cDNA library for interactors of atToc159G, atToc132G and atToc132AG baits. *S. cerevisiae* strain NMY51 was transformed with Toc159G, Toc132G or Toc132AG bait constructs and a split-ubiquitin yeast two-hybrid assay was performed utilizing positive, negative and empty prey plasmids. Optimization of the screening stringency was achieved through pilot screening, which involved large scale transformation of bait pre-transformed yeast with empty library vector. Selection of positive clones was conducted on the quadruple dropout media supplemented with 3-aminotrizole. The colonies that grew on the selective media were re-plated on the same stringent selective medium. Prey plasmids were re-isolated from the putative positive clones. Two degrees of selection (i.e. individual retransformation with respective baits and bait dependency test) were followed by sequencing and BLAST analysis to confirm the interactions and simultaneously identify the interactors.

To identify preproteins that may interact with the atToc159 family of receptors, we generated two cDNA libraries with mRNA isolated from ten-day old Arabidopsis seedlings. The two cDNA libraries were *i)* a random-primed library producing prey proteins fused to the N-terminus of NubG (prey-NubG) in the pR3-C vector and *ii)* an oligo-dT primed library producing prey proteins fused to the C-terminus of NubG (NubG-prey) in the pR3-N vector. Each library had an average complexity of 2×10^6^ transformants and an average insert size of 1.2 kb (data not shown). NMY51 yeast strains harbouring the Toc159G, Toc132G or Toc132AG bait plasmids were transformed with both of the cDNA libraries. Transformants were first selected on plates lacking leucine, tryptophan, histidine and adenine (SD-LWHA) to select for protein interactions on the basis of the activation of the *ADE2* and *HIS3* reporter genes. Background growth in library transformation screening due to leaky *HIS3* gene expression was suppressed by adding 3-aminotriazole (3AT) to a concentration of 20 mM for the Toc159G bait, 40 mM for Toc132G bait, and 50 mM for Toc132AG bait library screening plates. Although the Toc159G bait was first screened with the random-primed Arabidopsis cDNA library, the numbers of transformants were very low and significantly fewer positive clones were identified than when the oligo-dT-primed library was used. The optimized screening procedure for all three bait proteins with the Arabidopsis cDNA library is illustrated in Figure 2. Twenty-eight µg of library DNA plasmid was used for each screen with transformation efficiency above 2.5×10^5^ clones/µg DNA for all of the library transformations, which is sufficient enough to cover the cDNA libraries multiple times. Approximately 300–400 clones that grew on the SD-LWHA/3-AT plates from each of the Toc159G, Toc132G and Toc132AG bait screens were re-plated on the SD-LWHA/3-AT medium. From those strains growing after 3–4 days on SD-LWHA/3-AT selective media, prey plasmids were isolated, transformed into *E. coli* and re-isolated; the inserts were analyzed by restriction digest. The prey plasmids carrying an insert were further tested for bait-prey interactions through pairwise yeast two-hybrid interaction studies using Toc159G, Toc132G or Toc132AG as bait and each individual prey. Prey plasmids from positive clones were sequenced using vector specific primers, and were identified using Basic Local Alignment Search Tool (BLAST) searches. Prey plasmids from positive clones were also re-tested for bait dependency by carrying out pairwise bait-prey interaction tests with an unrelated control bait plasmid pTSU2-APP. Protein interactions, indicated by yeast growth on stringent selective media, were further confirmed by assessing expression from the *LacZ* reporter gene using qualitative β-Gal assays (i.e. X-Gal filter assay as shown in Figure S3). The extracted prey protein interactors are listed in Tables 1 and 2.

**Table 1:** List of plastid proteins with a non-photosynthetic function identified as interactors with atToc159G-, atToc132G- and atToc132AG-domain bait proteins in the split-ubiquitin yeast two-hybrid screen.

Screening Bait^2^					
Name of Gene	Accession No.	cTP^1^	atToc159G	atToc132G	atToc132AG
Plastidial Dihydrolipoamide Acetyltransferase (PDA)	AT1G34430	48	**+**		
Acyl Carrier Protein 2 (ACP 2)	AT1G54580	51	**+**		
Chaperonin 60 Beta (Cpn60-beta-2)	AT1G55490	53	**+**		
VQ motif-containing protein (VQ motif)	AT2G22880	57	**+**		
Outer Plastid Envelope Protein 16-1 (AtOEP16-1)	AT2G28900	18	**+**	**+**	
ThiaminC (THIC)	AT2G29630	37	**+**		
Thioredoxin M-Type 4 (TRX-M4)	AT3G15360	82	**+**		
AIG2-like avirulence induced gene family protein (AIG2-like)	AT3G28940	36	**+**		**+**
Copper Chaperone (CCH)	AT3G56240	24	**+**		
Cytochrome b561-2 (ACYB2, Cyt b561-2)	AT4G25570	6	**+**	**+**	
Small Subunit Ribosomal Protein 16 (SSR16)	AT4G34620	22	**+**		
Rhomboid-Like Protein 11 (AtBL11)	AT5G25752	49	**+**		
Membrane-associated progesterone binding protein 3 (ATMAPR3)	AT3G48890	51	**+**		
Glucose-6-phosphate transmembrane transporter (GPT1)	AT5G54800	64	**+**		
Thioredoxin F-type 1 (Trx F1)	AT3G02730	57		**+**	
Chloroplast chaperonin 10 (Cpn10-2)	AT2G44650	39		**+**	
Ferretin 1 (FER1)	AT5G01600	47		**+**	
Tryptophan synthase, beta subunit 1 (TSB1)	AT5G54810	52		**+**	
Lipid-transfer protein (LTP)	AT2G45180	81		**+**	
Protein LURP-one-related 15 Protein (LURP-1)	AT5G01750	44		**+**	
Metallo-beta-lactamase family protein (MBL Family P)	AT4G33540	45		**+**	
Glutathione S-transferase PHI 2 (GSTF2)	AT4G02520	33		**+**	
ATPase, F0 complex, subunit B/B’ (CFO-II – atpG, PDE334)	AT4G32260	74		**+**	
Translationally controlled tumor protein (TCTP)	AT3G16640	4		**+**	
Fe superoxide dismutase 1 (FSD1)	AT4G25100	8		**+**	
Small GTP-binding protein (GTP Binding)	AT5G08650	51		**+**	
Lactoylglutathionelyase (LL)	AT1G08110	2		**+**	
THI1 -involved in thiamine synthesis (vitamin B) (ARA6)	AT5G54770	45		**+**	
Fatty acid desaturase 6 (FAD6)	AT4G30950	78		**+**	
Geranylgeranyl reductase (GGR)	AT4G38460	33		**+**	**+**
Sugar transporter EDR6-like 7 (EDR6-L7)	AT2G48020	53		**+**	**+**
MPBQ/MSBQ methyl transferase (APG1, E37)	AT3G63410	51		**+**	**+**
Cysteine synthase (OASA1)	AT4G14880	20			**+**
ATP carrier protein 1 (AAC1)	AT3G08580	60			**+**
CCR-like protein (CCL)	AT3G26740	41			**+**
5′-Adenylylsulfate reductase 2 (APR2)	AT1G62180	66			**+**
Plastid transcriptionally active 4 (PTAC4)	AT1G65260	66			**+**
NADH dehydrogenase (ubiquinone) 1 alpha−subcomplex 5 (UQ Alp5)	AT5G52840	10			**+**
Putative 3-dehydroquinate synthase (3 DHQS)	AT5G66120	13			**+**
GLNB1-like protein (GLB1)	AT4G01900	61			**+**
Dehydrin family protein (DFP)	AT1G54410	20			**+**

1 cTP denotes the length of the (predicted) chloroplast transit peptide.

2 ‘+’ denotes which bait proteins each prey protein interacted with.

**Table 2:** List of plastid proteins with a photosynthetic function identified as interactors with atToc159G-, atToc132G- and atToc132AG-domain bait proteins in the split-ubiquitin yeast two-hybrid screen.

Screening Bait^2^					
Name of Gene	Accession No.	cTP^1^	atToc159G	atToc132G	atToc132AG
PGR5-Like A (PGRL1A)	AT4G22890	60	**+**		
Photosystem I subunit D-2 (PSAD-2)	AT1G03130	43	**+**	**+**	**+**
Photosystem II Subunit P-1 (Psb-1)	AT1G06680	31	**+**	**+**	**+**
Plastocyanin 2 (PETE2)	AT1G20340	52	**+**		
Photosystem II Subunit S (NPQ4, PsdS)	AT1G44575	59	**+**		
Photosystem II 5 kD protein (psbTn-2)	AT1G51400	33	**+**		
Photosystem II subunit X (PSBX)	AT2G06520	58	**+**		**+**
Photosystem I subunit E-2 (PSAE-2)	AT2G20260	46	**+**		
Cytochrome b6f complex subunit M (petM)	AT2G26500	84	**+**	**+**	
Photosystem II light harvesting complex gene B1B2 (LHCII-1.5)	At2G34420	37	**+**		**+**
Photosystem I P Subunit (PSI-P)	AT2G46820	45	**+**		
Light-Harvesting Chlorophyll-Protein Complex I Subunit A4 (LHCA4)	AT3G47470	49	**+**	**+**	**+**
Photosystem I subunit D-1(PSAD-1)	AT4G02770	44	**+**	**+**	
Photosystem I subunit L (PSAL)	AT4G12800	50	**+**	**+**	
Photosystem I reaction centre subunit IV (PSAE-1)	AT4G28750	44	**+**		
Light harvesting complex photosystem II (LHCII-4.1)	AT5G01530	40	**+**		**+**
RuBisCO small subunit 3B (RBCS-3B)	AT5G38410	54	**+**	**+**	**+**
RuBisCO small subunit 1B (RBCS-1B)	AT5G38430	54	**+**	**+**	
Light-Harvesting Chlorophyll B-Binding Protein 3 (LHCII-3)	AT5G54270	22	**+**		
Ferredoxin-NADP(+)-oxidoreductase 1 (FNR1)	AT5G66190	64	**+**		
Light Harvesting Complex Of Photosystem II 5 (LHCII-5)	AT4G10340	48	**+**	**+**	**+**
Chlorophyll A/B binding protein 1 (CAB1)	AT1G29930	23		**+**	**+**
Photosystem I light harvesting complex gene 3 (LHCI-3)	AT1G61520	48		**+**	**+**
Photosystem I reaction center subunit PSI-N (PSA N)	AT5G64040	81		**+**	
Photosystem I light harvesting complex gene 2 (LHCI-2.1)	AT3G61470	44		**+**	**+**
Photosystem I light harvesting complex gene 1 (LHCI-1-1)	AT3G54890	35		**+**	**+**
Rubisco small subunit 2b (RBCS-2B)	AT5G38420	54		**+**	**+**
Protochlorophyllide oxidoreductase B (POR B)	AT4G27440	43		**+**	
Light-harvesting chlorophyll-protein complex II subunit B1 (LHB1B1)	AT2G34430	23		**+**	**+**
Light harvesting complex photosystem II subunit 6 (LHCII-6)	AT1G15820	46		**+**	
Photosystem II light harvesting complex gene 2.1 (LHCII2.1)	AT2G05100	30		**+**	**+**
Photosystem I subunit F (PSAF)	AT1G31330	32		**+**	
Violaxanthin Deepoxidase (VDE, NPQ1)	AT1G08550	82		**+**	
Photosystem II light harvesting complex protein 2.3 (LHCII-2.3)	AT3G27690	28			**+**
Low PSII accumulation 3 protein (LPA3)	AT1G73060	58			**+**
Oxygen-evolving enhancer protein 3-2 (PSBQ-2)	AT4G05180	48			**+**
Photosystem I subunit G (PSAG)	AT1G55670	59			**+**
Photosystem II subunit T (PsbTn)	AT3G21055	69			**+**
Ferredoxin-thioredoxin reductase subunit A (FeThRed_B)	AT2G04700	31			**+**
Photosystem II reaction center PSB28 protein (PSB28)	AT4G28660	49			**+**

1 cTP denotes the length of the (predicted) chloroplast transit peptide.

2 ‘+’ denotes which bait proteins each prey protein interacted with.

### Toc159G-domain Interacting Proteins Identified using the Split-ubiquitin System

Identification of proteins that interact with the Toc159 G-domain was achieved by performing independent screens of the random-primed and oligo-dT-primed cDNA libraries, as described above. The screens using the Toc159G bait yielded a total of 70 positive clones representing genuine interactions, which were subsequently confirmed by pairwise transformations and a quantitative β-Gal assay (Figure S2-3). Overall, the two screens revealed a total of 35 unique putative prey proteins that interacted with Toc159G (Tables 1 and 2). Using information available in the Plant Proteome DataBase, PPDB [74] and the AT_CHLORO database [75], prey proteins were confirmed to be plastid proteins. For those proteins that were annotated as putative plastid proteins, the presence of a predicted TP was confirmed using ChloroP. Furthermore, interacting prey proteins were divided based on functional differences. Specifically, proteins were grouped based on having a non-photosynthetic (Table 1) or photosynthetic (Table 2) function. The interacting preproteins with a non-photosynthetic function (Table 1) included an array of divergent proteins involved in various essential cellular processes such as *i)* early embryo development (Plastidial Dihydrolipoamide Acetyltransferase and Glucose-6-phosphate transmembrane transporter), *ii)* biosynthetic pathways (Acyl Carrier Protein 2 ThiaminC, Rhomboid-Like Protein 11), *iii)* environmental stress signaling (VQ motif-containing protein), *iv)* seed development (Outer Plastid Envelope Protein 16-1), *v*) protein folding (Chaperonin 60 Beta), *vi*) redox reactions (Membrane-associated progesterone binding protein 3), and *vii*) redox regulation (Thioredoxin M-Type 4) (Table S1). Interestingly, one of the interacting proteins that was identified, small Subunit Ribosomal Protein 16, is a mitochondrial rps16 that can target to chloroplasts as well as mitochondria [76]. A subset of interactors that were revealed in the screen were uncharacterised chloroplast proteins, namely, AIG2-like (avirulence induced gene) family protein [77], copper chaperone and Cytochrome b561-2 [78], which are predicted to be involved in various essential non-photosynthetic processes. The predicted length of the N-terminal transit peptides was longer than 40 amino acids for almost all interacting preproteins except for ThiaminC (37 aa), AIG2-like family protein (36 aa), Copper Chaperone (24 aa) and the dual-targeted Small Subunit Ribosomal Protein 16 (22 aa). Only two of the non-photosynthetic interacting proteins, Outer Plastid Envelope Protein 16-1 and Cytochrome b561-2 lacked a predicted canonical chloroplast transit peptide.

The interacting proteins with photosynthetic functions (Table 2) mainly consisted of proteins belonging to *i)* Photosystem I (i.e. Photosystem I subunit D-2, Photosystem I subunit E-2, Photosystem I P-Subunit, Light-Harvesting Chlorophyll-Protein Complex I Subunit A4, Photosystem I subunit D-1, Photosystem I subunit L, Photosystem I reaction center subunit IV), *ii)* Photosystem II (i.e. Photosystem II Subunit P-1, Photosystem II Subunit S, Photosystem II 5 kD protein, Photosystem II subunit X, Photosystem II light harvesting complex gene B1B2, Light-Harvesting Chlorophyll B-Binding Protein 3, Light Harvesting Complex of Photosystem II 5, *iii)* electron transfer components (i.e. PGR5-Like A, Plastocyanin 2, Cytochrome b_6_ *f* complex subunit M, Ferredoxin-NADP(+)-oxidoreductase 1, and *iv)* the RuBisCO small subunit 1B/3B (Table S1). All of the interactors with photosynthetic function included a known transit peptide typical of nucleus-encoded chloroplast preproteins.

### Interactions of Toc159G-interacting Prey Proteins with Toc132G and Toc132AG Bait Proteins

**Figure 3:**
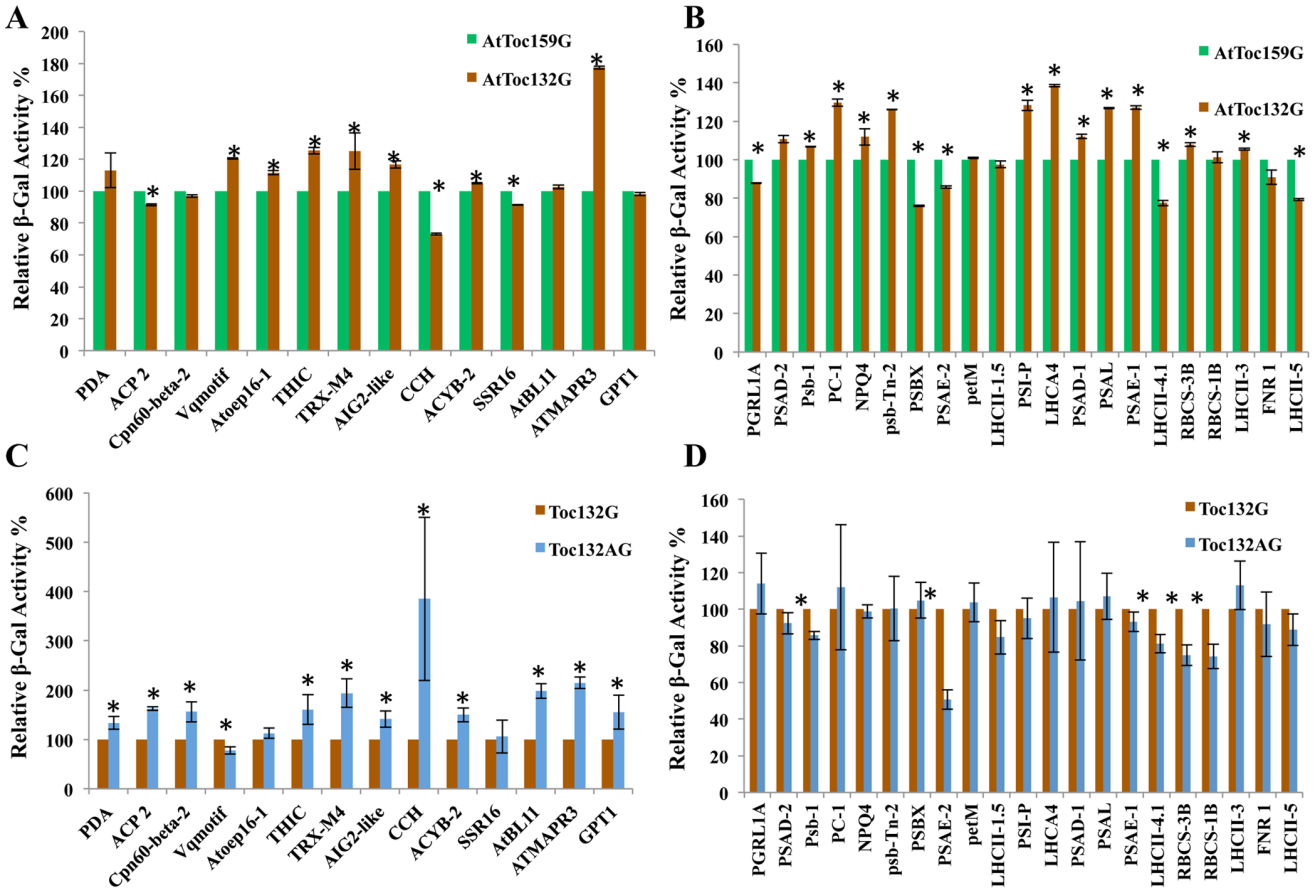
Comparative strengths of protein–protein interactions as determined by a quantitative β-galactosidase assay for prey proteins originally isolated from the cDNA library using the atToc159 G-domain as the bait. (A–D) Relative enzymatic activity of β-Gal in extracts from *S. cerevisiae* strain NMY51 that expressed atToc159G, atToc132G or atToc132AG bait and also carried NubG-Prey construct as indicated in Table 1 and 2. Yeast cells were grown to A_546_ of ∼0.8 in SD (−Leu, −Trp) medium at 30°C, followed by measurements as described in material and methods, and were quantified according to the following formula: Activity = 1,000×OD_615_/V×t×OD_546_, were V is the volume of assay and t is the time of incubation, for β-Gal activity in cell extracts. The measured activity was normalised to that of mock co-transformed strains expressing atToc159G, atToc132G or atToc132AG baits and empty vector, pR3-N, for respective interactions. A relative β-Gal activity of 100% was arbitrarily assigned to the atToc159G bait containing (A–B) and atToc132G bait containing (C–D) pairwise interactions. The experiments were performed in triplicate and repeated at least twice. Error bars indicate ±SD. Values marked with asterisks are significantly different (Student’s t-test; P≤0.05). (A) Interaction in yeast co-expressing atToc159G or atToc132G bait and non-photosynthetic prey interactors identified in atToc159G screening (Table 1). (B) Interaction in yeast co-expressing atToc159G or atToc132G bait and photosynthetic prey interactors identified in atToc159G screening (Table 2). (C) Interaction in yeast co-expressing atToc132G or atToc132AG bait and non-photosynthetic prey interactors identified in atToc159G screening (Table 1). (D) Interaction in yeast co-expressing atToc132G or atToc132AG bait and non-photosynthetic prey interactors identified in atToc159G screening (Table 2).

To compare the ability of the preproteins that were found to interact with Toc159G to also interact with atToc132, individual pairwise split-ubiquitin yeast two-hybrid interaction assays were performed using atToc132G and atToc132AG as the baits. Positive interactors were selected on the stringent selective SD-LWHA media and the strength of the interactions was evaluated though a quantitative β-Gal assay. All of the prey proteins identified in the 159G library screen were also positive interactors in the interaction assays using Toc132G and Toc132AG as the bait. The comparative strength of the interactions for most of the non-photosynthetic interacting prey proteins was similar for both Toc159G and Toc132G baits (Figure 3A). Two of the interactors, THIC and TRX-M4, exhibited a small, but significantly stronger interaction with Toc132G as compared to Toc159G, based on the quantitative β-Gal assay (i.e. the relative β-Gal activity was 25.3% higher for THIC and 25.0% higher for TRX-M4, when 132G was the bait as compared to 159G) (Figures. 3A). The strength of the interaction between ATMAPR3 and Toc132G was much higher (i.e. 77.5% higher) than that between ATMAPR3 and Toc159G (Figure. 3A). The opposite result was observed for the CCH prey, which demonstrated a noticeably stronger interaction with Toc159G than with Toc132G (the relative β-Gal activity was 27% lower when 132G was the bait as compared to 159G, Figure. 3A).

Inclusion of the A-domain of atToc132 together with the G-domain (Toc132AG) in the bait protein resulted in a noticeable increase in the strength of the interaction with non-photosynthetic prey proteins as compared to the Toc132 G-domain alone that didn’t include the A-domain (Toc132G) (Figure 3C). For example, an increase in the strength of the interaction with Toc132AG as compared to Toc132G was observed for PDA (33.6%), ACP2 (62.2%), Cpn-beta-2 (55.8%), THIC (60.6%), TRX-M4 (93.8%), AIG2-like (41.3%), ACYB-2 (49.6%), ATBL11 (98%), ATMAPR3 (114.6%) and GPT1 (55.2%) (the relative increase in strength of the interaction is noted in parentheses). Of particular note is the large and significant increase in the strength of the interaction (284.7%) that was observed for Copper chaperone when the A-domain was included in the bait (i.e. Toc132AG as compared to Toc132G). AtOEP16-1 and SSR16 were the only two proteins among the non-photosynthetic interactors that did not show any significant difference in their affinity for atToc132 whether the A-domain was present or absent (Figure. 3C).

Differences among the three bait proteins were also observed for the strength of interaction with the photosynthetic prey proteins. Only five of the photosynthetic prey proteins, PGRL1A (13%), PSBX (24%), PSAE-2 (14.4%), LHCII4.1 (23%) and LHCII-5 (21%) exhibited a noticeably stronger interaction with Toc159G as compared to Toc132G (the numbers in parenthesis indicate the relative increase in the strength of interaction with Toc159G as compared to Toc132G) (Figure 3B). On the other hand, Psb-1 (−6.7%), PETE2 (−29.6%), NPQ4 (−11.9%), Psb-Tn-2 (−26.1%), PSI-P (−28.2%), LHCA4 (−38.5%), PSAD1 (−12.15%), PSAL (−26.8%), PSAE-1 (−27.1%), RBCS-3B (−7.84%) and LHCII-3 (−5.52%) interacted more strongly with Toc132G as compared to Toc159G (the numbers in parenthesis indicate the relative decrease in the strength of interaction with Toc159G as compared to Toc132G) (Figure 3B). The strength of the interactions between all of the remaining photosynthetic prey proteins investigated was similar for the Toc159G and Toc132G baits (Figure 3B). The strength of interactions for the same set of interactors with Toc132AG was comparable to the interaction with Toc132G (Figure 3D). Only five of the photosynthetic interactors showed reduced affinity for Toc132AG as compared to Toc132G. Specifically, the quantitative β-Gal assay of three independent experiments revealed a reduction in the relative strength of interaction with Psb-1, PSAE-2, LHCII-4.1, RBCS-3B and RBCS-1B by 15%, 50%, 18.9%, 25.1% and 25.8%, respectively.

### Toc132G-interacting Proteins Identified using the Split-ubiquitin System

Owing to the preprotein binding properties of the G-domain of the Toc159 receptor family, revealed in earlier *in vitro* studies [19], we carried out a split-ubiquitin yeast two-hybrid screen of our entire Arabidopsis cDNA library using Toc132G as the bait. Due to the low number of positive clones obtained when screening the random-primed cDNA library using the Toc159G bait, we restricted our library screening with the Toc132G bait to the oligo-dT primed cDNA library. The screen revealed a total of 80 positive clones, representing 41 different prey proteins, as putative interactors of Toc132G. Eleven of the 41 putative interactors were also identified when screening the library with the Toc159G bait (compare Tables 1 and 2). Analysis of the clones using the Plant Proteome DataBase, [74] and the AT_CHLORO database [75], revealed them to encode full-length chloroplast precursor proteins. As with the interactors identified when screening the library with 159G, the interactors identified in the screen using Toc132G were also grouped according to whether they had a photosynthetic or non-photosynthetic function. Although the non-photosynthetic interactors included both stromal and membrane localized proteins, the photosynthetic interactors were predominantly membrane localized according to annotations. Interestingly, only two of the 11 proteins that were identified in both library screens (i.e. using Toc132G and Toc159G as the bait) were non-photosynthetic proteins (Outer Plastid Envelope Protein 16-1 and Cytochrome b561-2, Table 1). The other proteins identified in the screen using 132G as the bait that were characterized as having a non-photosynthetic function fall into many different functional classes, such as proteins involved in *i)* carbohydrate metabolism (Thioredoxin F-type 1 and lactoylglutathione lyase), *ii)* stress response (Ferretin 1, Glutathione S-transferase PHI 2 and Fe superoxide dismutase 1), *iii)* biosynthetic pathways (Tryptophan synthase beta subunit 1, THI1-involved in thiamine synthesis, Fatty acid desaturase 6, Geranylgeranyl reductase and MPBQ/MSBQ methyl transferase), *iv)* transport processes (Lipid-transfer protein, ATPase F_0_ complex, subunit B/B’ and sugar transporter EDR6-like 7), *v)* protein folding (Chloroplast chaperonin 10), *vi)* cell growth regulation (Translationally controlled tumor protein), *vii)* translation processes (Small GTP-binding protein), and *viii)* catalytic activity (Metallo-beta-lactamase family protein (Table S1). One of the interactors, Protein LURP-one-related 15 Protein, is a chloroplast-localised protein with putative non-photosynthetic functional affiliation [77]. Most of the proteins in the non-photosynthetic group carried a strongly predicted N-terminal transit peptide typical of chloroplast precursor proteins. Only three of the interactors, namely, Translationally controlled tumor protein, Fe superoxide dismutase 1, and lactoylglutathione lyase lacked a predicted, canonical N-terminal transit peptide for chloroplast localization (Table 1).

Of the 11 proteins that were common to the lists of proteins identified when screening the library with 159G and 132G, 9 had photosynthetic functions (Table 2). The additional photosynthetic related interacting proteins that were identified in the Toc132G screen (Table 2) predominantly consist of proteins of Photosystem I, Photosystem II, the chlorophyll biosynthetic pathway, the xanthophyll cycle, and RuBisCo small subunit 2B. All of the interacting partners with a photosynthetic function carried a predicted N-terminal transit peptide for chloroplast localization (Table 2).

### Ability of Prey Proteins Identified in the Toc132G Library Screen to Interact with Toc159G and Toc132AG Baits

**Figure 4:**
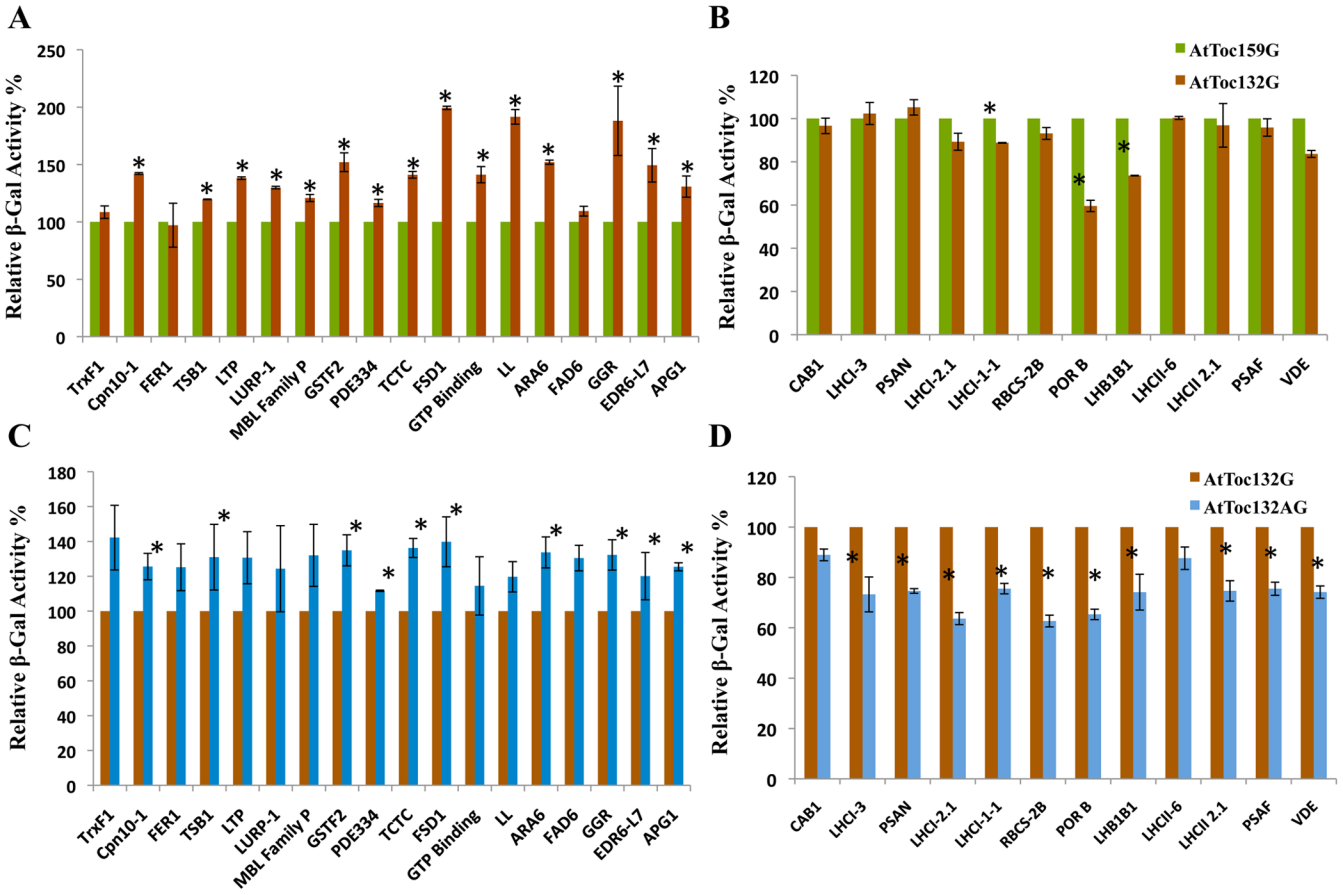
Comparative strengths of protein–protein interactions as determined by a quantitative β-galactosidase assay for additional prey proteins isolated from the screen using atToc132G as the bait. (A–D) Relative enzymatic activity of β-Gal in extracts from *S. cerevisiae* strain NMY51 that expressed atToc159G, atToc132G or atToc132AG baits and also carried the NubG-Prey construct as indicated in Table 1 and 2. Yeast cells were grown to A_546_ of ∼0.8 in SD (−Leu, −Trp) medium at 30°C, followed by measurements as described in Materials and Methods, and were quantified according to the following formula: Activity = 1,000×OD_615_/V×t×OD_546_, were V is the volume of assay and t is the time of incubation, for β-Gal activity in cell extracts. The measured activity was normalised to that of mock co-transformed strains containing atToc159G, atToc132G or atToc132AG bait and empty vector, pR3-N, for respective interactions. A relative β-Gal activity of 100% was arbitrarily assigned to the atToc159G bait containing (A–B) and atToc132G bait containing (C–D) pairwise interactions. The experiments were performed in triplicate and repeated at least twice. Error bars indicate ±SD. Values marked with asterisks are significantly different (Student’s t-test; P≤0.05). (A) Interaction in yeast co-expressing atToc159G or atToc132G bait and additional non-photosynthetic prey interactors identified in the screen using atToc132G as the bait (Table 1). (B) Interaction in yeast co-expressing atToc159G or atToc132G bait and additional photosynthetic prey interactors identified in the screen using atToc132G as the bait (Table 2). (C) Interaction in yeast co-expressing atToc132G or atToc132AG bait and additional non-photosynthetic prey interactors identified in the screen using atToc132G as the bait (Table 1). (D) Interaction in yeast co-expressing atToc132G or atToc132AG bait and additional non-photosynthetic prey interactors identified in the screen using atToc132G as the bait (Table 2).

Of the 41 unique prey proteins identified in the library screen using Toc132G as the bait, 11 were also identified in the screen using Toc159G (compare Tables 1 and 2). To compare the ability of the other 30 prey proteins identified in the library screen using 132G as the bait to interact with other baits, individual pairwise split-ubiquitin yeast two-hybrid assays were performed. The clones were screened on the highly stringent SD-LWHA/3-AT media and the strength of interactions were measured using the quantitative β-Gal assay for each clone co-expressing respective bait and prey proteins. All 30 prey proteins tested that had been uniquely identified in the 132G bait screen also interacted with the other two baits (i.e. Toc159G and Toc132AG). Comparison of the relative strength of the interactions of the non-photosynthetic prey proteins (Table 1) with Toc159G and Toc132G revealed that most of the non-photosynthetic prey proteins had a relatively higher affinity for 132G. Ten of the proteins were observed to have a significantly stronger interaction for 132G than for 159G (Cpn10-1 (42.1%), LTP (38.1%), GSTF2 (52%), TCTC (41%), FSD1 (99.5%), GTP Binding (41.1%), LL (91.5%), ARA6 (52%), GGR (88%) and EDR6-L7 (49.3%) (numbers in parentheses represent the relative increase in strength of the interaction with 132G as compared to 159G). TSB1 (19.6%), LURP-1 (29.9%), PDE334 (16.5%) and APG1 (30.7%) had a smaller, but still comparably higher affinity for Toc132G as compared to Toc159G. Three of the interactors (TrxF1, FER1 and FAD6) were observed to have similar affinity for 132G and 159G in this assay (Figure 4A).

The β-Gal assay was also used to compare the relative strengths of the interactions of the same set of non-photosynthetic prey proteins with the two Toc132 bait variants, Toc132G and Toc132AG. The assay revealed that the presence of the A-domain increased the relative strength of the interaction between Toc132 and the non-photosynthetic proteins. Only 7 out of 18 prey proteins, namely, TrxF1, FER1, LTP, LURP-1, MBL Family P, GTP binding and FAD6, showed no significant difference in the strength of the interaction when the A-domain was present (Figure 4C). The difference in the strength of interaction of the photosynthetic prey proteins identified in the Toc132G screen for Toc159G and Toc132G was largely indistinguishable (Table 2). No significant difference in the affinity of Toc159G and Toc132G bait was observed for most of the prey proteins in this group (Figure 4B). Only 3 out of 12 photosynthetic interactors, showed higher affinity for Toc159G as compared to Toc132G. The quantitative β-Gal assay revealed a relatively stronger interaction of LHCI-1-1 (11.3% stronger), POR B (40.5% stronger) and LHB1B1 (26.4% stronger) with 159G as compared to when 132G was used as the bait. Interestingly, a decrease in affinity was observed for almost all of the photosynthetic-related prey proteins investigated in this assay when Toc132AG was used as the bait, as compared to when Toc132G was used (Figure 4D). A significantly lower affinity for Toc132AG than for Toc132G was observed for LHCI-3 (−26.8%), PSAN (−25.4%), LHCI-2.1 (−36.4%), LHCI-1-1 (−24.5%), RBCS-2B (−37.3%), POR B (−34.7%), LHB1B1 (−25.9%), LHCII 2.1 (−25.4%), PSAF (−25.5%) and VDE (−29.9%) (the numbers in parenthesis indicate the relative strength of the interaction for Toc132AG compared to Toc132G). Only two of the photosynthetic interactors, CAB1 and LHCII-6 did not show any significant difference in their affinity toward Toc132G or Toc132AG (Figure 4D).

### Toc132AG-interacting Proteins Identified using the Split-ubiquitin System

Since the variable acidic N-terminal A-domains of Toc159 and Toc132 were recently reported to determine the selectivity for different nucleus-encoded preproteins [37], it was of particular interest to extend our library screen to include Toc132AG as the bait as a way to examine the inherent selectivity potentially conferred by the A-domain. The library was screened as described for the Toc132G bait, to identify potential Toc132AG interactors. 67 positive clones representing a total of 35 different protein-encoding genes were identified (Table 1 and 2). Based on the Plant Proteome DataBase [74] and AT_CHLORO database [75], the 35 clones were confirmed to encode full-length chloroplast localized precursor proteins. Thirteen of the 35 clones encoded proteins with non-photosynthetic related functions (Table 1), whereas the remaining 22 clones encoded photosynthetic proteins (Table 2). Among the non-photosynthetic preproteins, only one prey protein (i.e. AIG2-like family protein) was also identified in the library screen using Toc159G as the bait. Similarly, only 3 of the 35 interactors (Geranylgeranyl reductase, sugar transporter EDR6-like 7 and MPBQ/MSBQ methyl transferase) were also identified in the screen using Toc132G as the bait (Table 1 and 2). As with the other screens, the non-photosynthetic interactors identified in the Toc132AG screen included proteins associated with various essential biological processes, such as *i)* biosynthetic pathways (Cysteine synthase and putative 3-dehydroquinate synthase), *ii)* membrane transport (ATP carrier protein 1), *iii)* circadian clock regulation (CCR-like protein), *iv)* sulphate assimilation (5′-Adenylylsulfate reductase 2), *v)* membrane biogenesis (Plastid transcriptionally active 4), *vi)* oxidation-reduction (NADH dehydrogenase (ubiquinone) 1 alpha subcomplex 5), *vii)* cell signaling (GLNB1-like protein) and *viii)* stress response (Dehydrin family protein) (Table S1). Almost all of the non-photosynthetic chloroplast protein interactors, except Cysteine synthase, NADH dehydrogenase (ubiquinone) 1 alpha subcomplex 5, and putative 3-dehydroquinate synthase, were predicted to carry a strong N-terminal transit peptide for chloroplast localization (Table 1).

Among the interactors identified in the Toc132AG screen with photosynthetic functions, 15 were also identified in the Toc159G and/or Toc132G screens (Table 2). The remaining prey proteins (that were only identified in the Toc132AG screen) belong predominantly to Photosystem II (i.e. Photosystem II light harvesting complex protein 2.3, Low PSII accumulation 3 protein, Oxygen-evolving enhancer protein 3–2, Photosystem II subunit T and Photosystem II reaction center PSB28 protein). The other photosynthetic proteins identified in the Toc132AG screen were Photosystem I subunit G and a photosynthetic enzyme regulator, ferredoxin-thioredoxin reductase subunit A (Table S1). All of the photosynthetic proteins identified in this screen carried a predicted cleavable chloroplast transit peptide (Table 2).

### Comparison of Toc132AG-interacting prey Proteins to Interact with Toc159G and Toc132G Baits using the Yeast Two-Hybrid System

**Figure 5:**
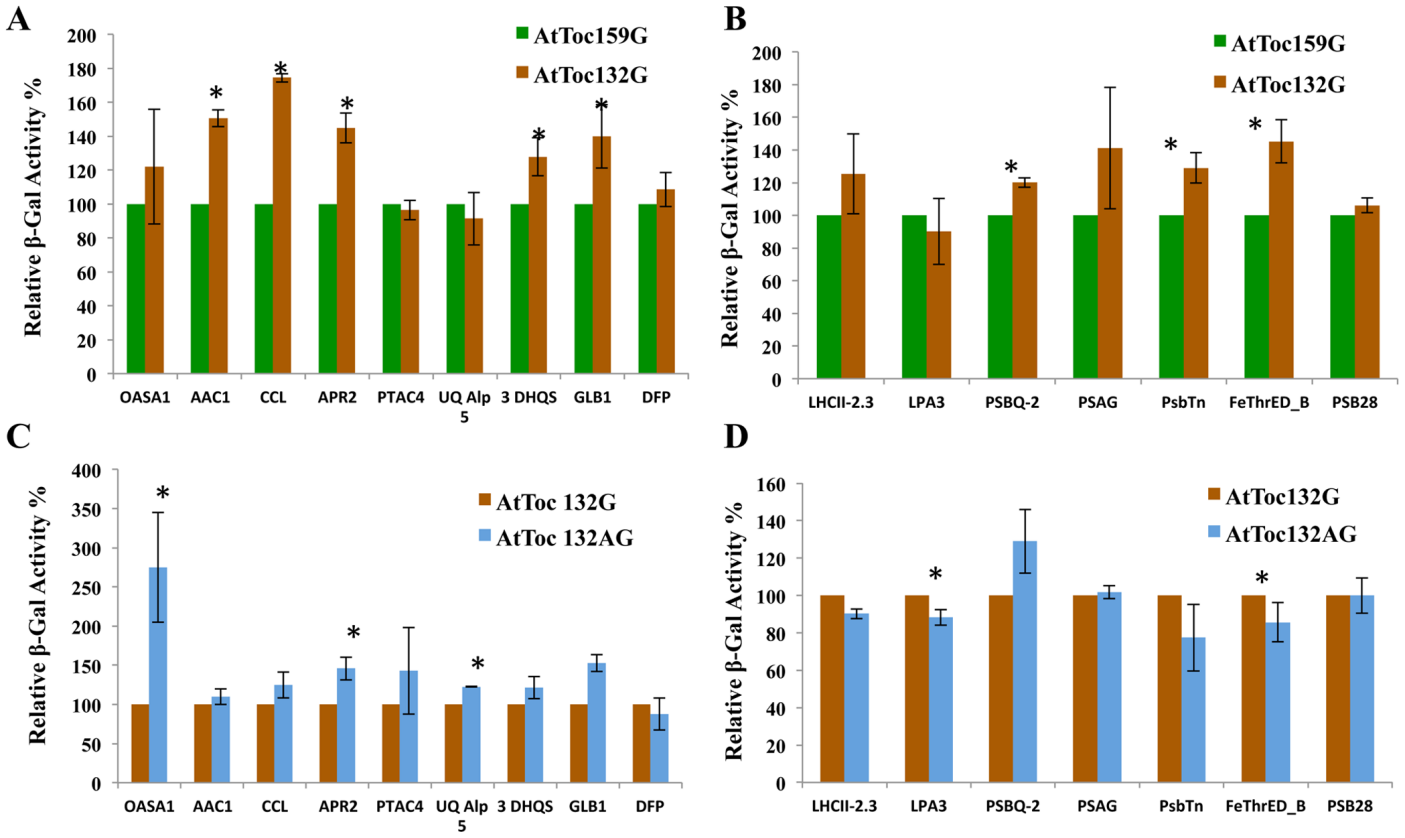
Comparative strengths of protein–protein interactions as determined by a quantitative β-galactosidase assay for additional prey proteins isolated from the screen using atToc132AG as the bait. (A–D) Relative enzymatic activity of β-Gal in extracts from *S. cerevisiae* strain NMY51 that expressed atToc159G, atToc132G or atToc132AG-domain bait and also carried NubG-Prey construct as indicated in Table 1 and 2. Yeast cells were grown to A_546_ of ∼0.8 in SD (−Leu, −Trp) medium at 30°C, followed by measurements as described in materials and methods, and quantified according to the following formula: Activity = 1,000×OD_615_/V×t×OD_546_, were V is the volume of assay and t is the time of incubation. For β-Gal activity in cell extracts, the measured activity was normalised to that of mock co-transformed strains containing atToc159G, atToc132G or atToc132AG bait and empty vector, pR3-N for respective interactions. A relative β-Gal activity of 100% was arbitrarily assigned to the atToc159G bait containing (A–B) and atToc132G bait containing (C–D) pairwise interactions. The experiments were performed in triplicate and repeated at least twice. Error bars indicate ±SD. Values marked with asterisks are significantly different (Student’s t-test; P≤0.05). (A) Interaction in yeast co-expressing atToc159G or atToc132G bait and additional non-photosynthetic prey interactors identified in the screen using atToc132AG as the bait (Table 1). (B) Interaction in yeast co-expressing atToc159G or atToc132G bait and additional photosynthetic prey interactors identified in the screen using atToc132AG screening (Table 2). (C) Interaction in yeast co-expressing atToc132G or atToc132AG bait and additional non-photosynthetic prey interactors identified in the screen using atToc132AG screening (Table 1). (D) Interaction in yeast co-expressing atToc132G or atToc132AG bait and additional non-photosynthetic prey interactors identified in the screen using atToc132AG screening (Table 2).

The interactions of the 16 prey proteins that were uniquely identified in the Toc132AG screen, with each of the 3 different baits were compared using individual pairwise split-ubiquitin yeast two hybrid assays as described above, and the relative strength of the interactions was measured using the quantitative β-Gal assay for each clone co-expressing the 3 baits. All 16 additional prey identified in 132AG screen also interacted with the other two baits (i.e. Toc159G and Toc132G). Among the non-photosynthetic interactors, 5 of the prey showed a significantly stronger interaction with Toc132G as compared to Toc159G (i.e. AAC1 (50.6%), CCL (74.4%), APR2 (44.7%), 3DHQS (27.8%) and GLB1 (39.8%); numbers in parentheses represent the relative increase in strength of the interaction with 132G as compared to 159G) (Figure 5A). No significant differences in the strength of interactions with Toc159G and Toc132G were observed for the remaining non-photosynthetic prey (Figure 5A). Presence of the A-domain fused to Toc132G (Toc132AG) in the bait, increased the affinity with 4 prey proteins, namely, OASA1 (175%), APR2 (24.5%), UQAlp5 (45.77) and GLB1 (52.8) (the relative increase in strength of the interaction is noted in parentheses; Figure 5C). No significance difference in the strength of interaction for Toc132G or Toc132AG was observed for the remaining non-photosynthetic interactors as is shown in Figure 5C.

Comparing the affinity of the additional 7 photosynthetic prey proteins identified in the Toc132AG screen, for Toc159G and Toc132G revealed a somewhat stronger interaction of PSBQ-2 (20%), PsbTn (28.9%) and FeThrED-B (45.2%) with Toc132G as compared to Toc159G. No significant difference in the affinity for Toc159G and Toc132G bait was observed for the remaining prey proteins (LHCII-2-3, LPA3, PSAG and PSB28) (Figure 5B). Extending our study to compare the strength of interaction for the same group of photosynthetic prey proteins for Toc132G and Toc132AG revealed no significant difference in the affinity of most of the interactors for either of these baits (Figure 5D). Only 2 out of 7 prey interactors, namely, LPA3 (−11.7%) and FeThrED-B (−15%) displayed a noticeably lower affinity for Toc132AG as compared to Toc132G (the numbers in parenthesis indicate the relative strength of the interaction).

### Analysis of Toc159AG- and Toc132AG-domain Interactions with the Transit Peptides of Representative Chloroplast Preproteins

Based on the previously hypothesized alternate import pathways for photosynthetic and non-photosynthetic preproteins and recent evidence that the A-domain of the atToc159 family of receptors contributes to the regulatory role in their preprotein recognition and/or selectivity [37], we examined the relative binding properties of atToc159AG and atToc132AG with transit peptides of representative photosynthetic preproteins, namely, Light-Harvesting Chlorophyll-Protein Complex I Subunit A4 (LHCA4) and Ferredoxin-NADP(+)-oxidoreductase 1 (FNR1). As we were unable to obtain a functional form of atToc159AG as a bait protein in *S. cerevisiae* NMY51, we employed a solid phase binding assay that was used previously to study the association of atToc159 receptors with Toc33/34 and transit peptides of chloroplast preproteins [19], [37].

**Figure 6:**
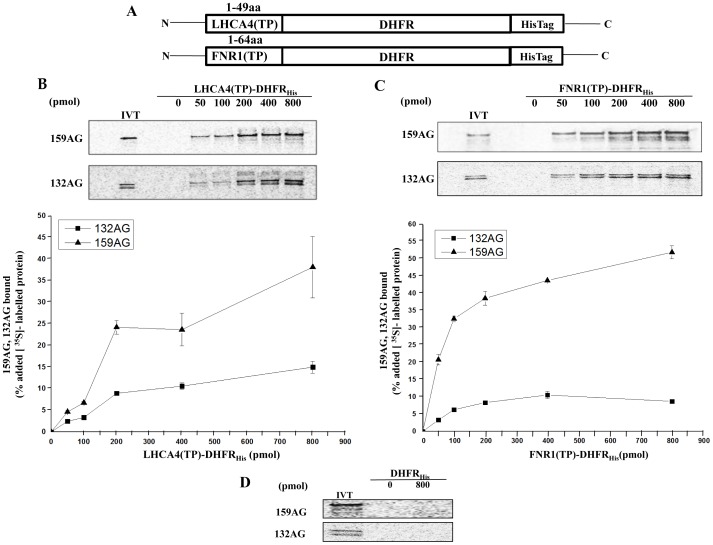
Direct binding of atToc159AG and atToc132AG to transit peptides (TPs) from two representative photosynthetic preproteins. (A) Schematic representation of the LHCA4(TP)-DHFR_His_ and FNR1(TP)-DHFR_His_ constructs with C-terminal histidine tags (His-Tag) used as baits in the binding reactions. The numbers refer to the amino acid numbers of the TPs from LHC4 and FNR. (B and C) Equal amounts of *in vitro* translated [^35^S]atToc132AG, or [^35^S]atToc159AG were incubated in the presence of GTP with the indicated amounts of immobilized hexahistidine-tagged LHCA4(TP)-DHFR_His_ (A) or FNR1(TP)-DHFR_His_ (B). Bound proteins were eluted and separated by SDS-PAGE, and detected in dried gels using a phosphorimager. Top panels of B and C show representative experiments of triplicates. Lane, IVT in each panel contains 10% of the *in vitro* translation product added to each reaction. The graphs show quantitative analysis of the triplicate binding experiments with SE bars. atToc159AG binds to LHCA4(TP)-DHFR_His_ and FNR1(TP)-DHFR_His_ at levels that are two-fold and five-fold higher than atToc132AG, respectively. The atToc132AG and atToc159AG do not significantly bind to 800 pmol of a negative control immobilized hexahistidine-tagged DHFR_His_ protein (D).

Recombinant forms of LHCA4 and FNR1 transit peptides fused to hexahistidine-tagged dihydrofolate reductase (Figure 6A) were expressed in *E. coli* (Figure S1B) and immobilized on a Ni^2+^-NTA matrix. Samples of the immobilized LHCA4(TP)-DHFR or FNR1(TP)-DHFR were incubated with equal amounts of *in-vitro* translated, ^35^S-labeled atToc159AG or atToc132AG, and the amount of bound receptor was determined by autoradiography as described in materials and methods. As shown in Figure 6B, both atToc159AG and atToc132AG bind in a dose-dependent manner to the LHCA4(TP)-DHFR. At the highest levels of LHCA4(TP)-DHFR tested (800 pmol) atToc159AG (38.2%) bound with more than twice the efficiency of atToc132AG (15%). Similarly, direct binding assays of the receptors with FNR1(TP)-DHFR recombinant proteins revealed that atToc159AG (51.9%) bound much more efficiently compared to atToc132AG (8.7%) at the highest amount of FNR1(TP)-DHFR tested (800 pmol; Figure 6C). These data suggest that atToc159AG binds to transit peptides of photosynthetic preproteins more efficiently than atToc132AG. Neither atToc159AG nor atToc132AG bound to a significant extent to the DHFR control lacking a transit peptide even at the highest level of bait tested (800 pmol), indicating that receptor binding was specific for the transit peptides (Figure 6D).

## Discussion

Identifying the protein interactome for the Toc159 family of chloroplast protein import receptors is a crucial step towards understanding preprotein targeting to the chloroplast. In this study, we used a large-scale qualitative and quantitative approach to investigate the intrinsic preprotein recognition properties of the Toc159 family of receptors from Arabidopsis. Previous studies on the structural and functional anatomy of Toc159 receptors have shed light on the initial recognition of preproteins by the import apparatus, and have led to the hypothesis that distinct import pathways exist for different functional classes of proteins [8], [9], [10], [11]. We attempted to expand the understanding of preprotein recognition and substrate specificity of distinct receptors by identifying and investigating the inherent binding characteristics of a large number of preprotein substrates for two members of this receptor family. To achieve this, we chose to use a yeast two-hybrid (Y2H) library screening strategy inasmuch as there was no *a priori* bias regarding which preproteins will be recognized by and interact with individual members of the Toc159 receptor family. As chloroplast development and biogenesis are active at the seedling stage, we generated cDNA libraries using RNA isolated from Arabidopsis at this early developmental stage to obtain maximum complexity of the genes represented in the screen. A typical Y2H requires the candidate proteins to be expressed in soluble form and targeted to the nucleus for potential interactions to be identified [69]. Owing to the advantages of using a more representative physiological environment (cytosol) and potential detection of both soluble and membrane protein interactions, we choose a split-ubiquitin membrane-based yeast two-hybrid system to search for interacting partners of the Toc159 receptors. Chemical-crosslinking and *in vitro* binding assays have shown the GTP-binding domain (G-domain) of Toc159 receptors to interact with preprotein transit peptide substrates [16], [17], [18], [19]. Therefore, we used bait proteins corresponding to the G-domains of atToc159 (Toc159G) and atToc132 (Toc132G) to screen the libraries. As preproteins are recognized by the receptors exclusively through their N-terminal transit peptides, we generated a separate random primed library with N-terminal prey (Prey-NubG library) along with a traditional oligo-dT primed library carrying C-terminal prey (NubG-Prey library) for the screens. With recent advances in the understanding of the A-domain and its regulatory role, we further extended our screen to include a Toc132AG construct as the bait, to test whether the presence of the A-domain may alter the types of preproteins that atToc132 interacts with, or the affinity with which it interacts with them. Although using a bait protein consisting of the A- and G-domains of atToc159 was the first choice for shedding light on the function of the A-domain, due to the pivotal role that atToc159 plays in chloroplast protein import [25], [26], [28], we were unable to express a functional version of an atToc159AG bait protein in the NMY51 yeast strain. Thus, we focused on the A-domain of atToc132 in order to better understand its role in recognition and binding of chloroplast preproteins.

Collectively, from a functional perspective, it is expected that most chloroplast preproteins are recognized and bound by at least one member of the Toc159 receptor family. Screening our Arabidopsis cDNA libraries using a highly stringent split-ubiquitin membrane-based yeast two-hybrid system with Toc159G bait identified 35 unique interactors representing approximately equal numbers of non-photosynthetic and photosynthetic chloroplast precursor proteins (Table 1 and 2). Contrary to our expectations, the oligo-dT primed, NubG-Prey cDNA library contributed almost all of the positive interactors identified in the screen using Toc159G as the bait. With an average insert size of 1.2 kb and complexity higher than 2×10^6^ transformants, we expected numerous interactors to also be identified from the random primed cDNA library (Prey-NubG library), as the TPs of plastid proteins should be on the N-terminus of these prey fusion proteins. The inefficiency of identifying positive interactors among the prey proteins produced by this library could be attributed to a defect in some *in vivo* compatibility,a modification in the receptor binding site(s) and/or because of (mis−)localization of the prey proteins produced by this library, rendering them unable to bind the bait proteins. For example, *in vitro* and *in vivo* studies in yeast using chimeric constructs consisting of the transit peptide of ribulose-1,5-bisphosphate carboxylase small subunit from *Chlamydomonas* and *Nicotiana sylvestris* fused to mouse cytosolic dihydrofolate reductase (DHFR) have shown “mistargeting” of the fusion protein to the mitochondria [79]. Similarly, the chloroplast β-barrel proteins Oep37 and Oep24 were exclusively localized to mitochondria when expressed in yeast, [80]. Likewise, it is also possible that some of the prey proteins expressed in our yeast system with free N-termini corresponding to chloroplast transit peptides (i.e. from the prey-NubG library), were targeted and imported into the mitochondria, rendering them unavailable to interact with the Toc159G bait during the screening process. Thus, we focused only on the oligo-dT primed, NubG-Prey cDNA library for the screens using Toc132G and Toc132AG baits. The screens identified 41 and 35 positive interactors for 132G and 132AG baits, respectively (Tables 1 and 2). With some repetition of interactors identified in the screens with different baits, we identified a total of 81 putative positive interactors using Toc159G, Toc132G or Toc132AG as the bait (Tables 1 and 2). With ∼2100 Arabidopsis chloroplast proteins encoded by nuclear genes, and the TOC/TIC complexes as the primary translocation pathway, we expected to identify a larger number of interactors in our screens. The relatively low number of interactors identified could be explained by several factors. For example, *in vitro* studies have demonstrated a direct interaction between the Toc159 G-domain and TPs [19], but it is entirely possible that the interaction involves other cellular components and events *in planta* that aren’t present in yeast cells [8], [13], [14], [30], [38]. It is also known that discrepancies in the strength of interactions can result from using a heterologous system such as yeast. Differences in the number and strength of interactions were observed for a basic leucine zipper (bZIP) protein in a plant-based system as compared to a yeast system, suggesting that additional important *in vivo* factors may govern the interactions in plants [81]. It is also important to consider that 3-aminotriazole (3AT) was included in our selection media (at concentrations ranging from 20–50 mM) in order to minimize the number of background colonies resulting from the leakiness of the *HIS3* gene. Such high concentrations of 3AT may have inhibited some true positive, transient, interactions between the Toc159 receptors and transit peptides, thereby reducing the number of interactors identified. The transient nature of interactions between chloroplast protein import receptors and TPs is also an important consideration. By definition, the interaction between preprotein TPs and chloroplast import receptors *in planta* must be transient in order to allow import to proceed. Assuming that the transient nature of interactions is maintained in a heterologous system such as yeast cytosol, it is possible that some TP-receptor interactions were missed under the stringent conditions used in our assays. Collectively, the low number of prey proteins identified could be attributed to masking of transient interactions due to the high stringency conditions used in our screening procedure, as well as the absence of potential plant-specific factors.

Our analysis identified approximately equal numbers of photosynthetic and non-photosynthetic chloroplast proteins as interaction partners for the G-domains of both atToc159 and atToc132. These results are consistent with previous *in vivo* and *in vitro* studies suggesting that the G-domains of the Toc159 receptors include a TP binding site [19], [21], but also suggest that these domains alone lack the ability to confer specificity towards photosynthetic or non-photosynthetic preproteins. These findings are also consistent with previous *in vivo* studies demonstrating the ability of the G-domains of the atToc159 and atToc132 receptors to partially complement *ppi2* mutant plants [21], [37], and with the notion that it is the variable A-domains of the Toc159 family of receptors that confer substrate specificity. In an effort to test the role of the A-domain, we re-screened the NubG-Prey library with a bait protein consisting of the G- and A-domains of atToc132. When the A-domain was included as part of the atToc132 bait construct (Toc132AG), the interactors still included both photosynthetic and non-photosynthetic proteins; in fact, slightly more photosynthetic proteins than non-photosynthetic proteins were found to interact with Toc132AG as compared to the atToc132 G-domain alone (Toc132G) (Table 1 and 2). The prevailing hypothesis regarding functional differentiation among the Toc159 family members, and the potential role of the A-domain in conferring the functional differences, predicted that the presence of the A-domain of atToc132 would have shifted the preprotein binding properties of this receptor such that it favoured an association with non-photosynthetic preproteins. That such a shift was not observed, and in fact the A-domain containing bait interacted with slightly more photosynthetic than non-photosynthetic proteins, suggests that the substrate specificity of the Toc159 family of receptors is not strictly defined by the functional classification of the preprotein cargo. While it’s possible that these findings were due to cytosolic differences between plants and yeast, the data suggest that atToc132 exhibits intrinsic binding affinity for both photosynthetic and non-photosynthetic preproteins. It is also possible that these data may, in part, be the result of an over-representation of mRNAs for photosynthetic proteins in the tissue that was used to prepare the cDNA library (developing seedlings). The earlier study that established the ability of Toc132GM to partially complement *ppi2* (atToc159 knockout) mutants, indicated that there is at least some overlap in the preprotein recognition function of atToc159 and atToc132 [37]. Inclusion of the A-domain of Toc159 fused to Toc132GM more effectively complemented the *ppi2* phenotype, indicating that the A-domain does play some role in the interaction of the receptor with precursor proteins. The data presented in the current study are consistent with the concept that the members of the Toc159 receptor family have different but overlapping substrate specificities.

We also evaluated the relative strength of the interaction between each of the interactors identified in the library screens and each of the three baits using a quantitative *in vitro* β-galactosidase assay [68], [69], [70]. In addition to evaluating the strength of interactions, these assays also served to provide independent confirmation that all of the preproteins identified in the Y2H screens interact with each of the bait proteins; in all cases the interaction was readily detectable (Figures 3, 4 and 5), therefore confirming that they represented genuine interactions. These data reaffirm the observations from our library screens and suggest that both atToc159 and atToc132 interact with all functional classes of preproteins *in vitro*. We were not able to identify any obvious physico-chemical characteristics of the TPs, such as the length of the (predicted) TPs, that correlated with differences observed in the strength of interaction with the different baits. Rather, the observed differences in strength of interaction appear to be preprotein-specific for each pair of interactors (Figures 3, 4 and 5). A more detailed analysis including TPs from a larger number of preproteins may reveal characteristics of TPs that are preferentially recognized by one receptor or another. Out of the 41 non-photosynthetic preprotein interactors investigated, 27 (∼66%) had a statistically higher affinity for Toc132G as compared to Toc159G (Figures 3A, 4A and 5A). Inclusion of the A-domain of Toc132G increased this receptor’s affinity for most of the non-photosynthetic interactors tested (Figures 3C, 4C and 5C); of note, is that the affinity for Toc132AG was significantly stronger for 6 of the non-photosynthetic preproteins (PDA, Cpn60-beta-2, AtBL11, GPT1, OASA1, UQ-Alp5), which earlier showed no difference in affinity for Toc159G or Toc132G (Figures 3A-C and 5A-C). Furthermore, our *in vitro* analysis indicates that there is a subset of non-photosynthetic preproteins whose intrinsic binding affinity may be similar for Toc159G and Toc132G/AG (i.e. TrxF1, FER1, FAD6, PTAC4, DFP). The difference in affinity of atToc132 for different non-photosynthetic proteins in the presence or absence of the A-domain points toward the possible existence of subsets of non-photosynthetic proteins that have variable affinities for the different receptors. An earlier study using chimeric proteins containing the TPs from pFd and pE1α suggested a regulatory function for the A-domain in determining the binding properties of atToc159 receptors [37]. Therefore, although the A-domain of atToc132 is reported not to interact directly with preprotein TPs [19], it appears to positively influence the preferential binding of non-photosynthetic preproteins, at least in some cases.

Comparison of the quantitative interaction data of Toc159G and Toc132G with the 40 photosynthetic proteins (Table 2) identified in the library screens reveals no significant difference between the binding efficiency of 18 interactors with both of the baits (Figures. 3B, 4B and 5B). Of the remaining 22 photosynthetic preprotein interactors, only 8 showed significantly higher affinity for Toc159G, whereas the other 14 proteins interacted more strongly with Toc132G. In the presence of the A-domain of atToc132 (i.e. for the Toc132AG bait) we observed a significant reduction in the affinity of this receptor for some of the photosynthetic interactors (Figures 3B-D, 4B-D and 5B-D). This suggests a negative regulatory role of the A-domain of atToc132 for the ability of this receptor to associate with photosynthetic preproteins. That the function of the atToc159 A-domain is to positively regulate the recognition of photosynthetic preproteins has been suggested by previous *in vivo* and *in vitro* studies [37]. Consistent with these previous studies, using two chimeric constructs containing TPs of representative photosynthetic proteins LHCA4 and FNR1, we observed higher efficiency of binding to Toc159AG as compared to Toc132AG in an *in vitro* binding assay (Figure 6). These data are consistent with a positive regulatory role of the atToc159 A-domain in the recognition of photosynthetic preproteins.

Thus using a membrane-based split ubiquitin yeast two-hybrid screening approach and an extensive pairwaise interaction assay we propose the possible existence of specific subsets of preproteins amongst the photosynthetic and non-photosynthetic classes, which have different affinities towards members of the Toc159 receptor family. Apart from suggesting a potentially antagonistic role for the atToc132 A-domain that might limit the binding of atToc132 to particular photosynthetic and non-photosynthetic proteins, we also substantiate for the first time that atToc132 can serve as a receptor by directly binding to chloroplast preproteins (via its G-domain). Recently it has been shown that different groups of preproteins vary in their import efficiency, which is regulated by the age of the chloroplast [24]. Import of both photosynthetic and non-photosynthetic preproteins is equally essential for proper chloroplast biogenesis. Contrary to the prevailing hypothesis that there are separate receptors for photosynthetic and non-photosynthetic chloroplast proteins, our data suggest that the specificities of atToc159 and atToc132 are largely overlapping. On the other hand, the presence of the A-domain did enhance the affinity of atToc132 for a subset of non-photosynthetic proteins, and seemed to reduce its affinity for some photosynthetic proteins. Overall, the data suggest that the family of Toc159 receptors have overlapping substrate specificities, but may exhibit some degree of specificity for particular preproteins, and that the specificity is conferred by the A-domain. The exact determinants of specificity may become more evident as a larger number of substrate proteins are identified. The ability of atToc132 (and all members of the Toc159 family of receptors) to bind both photosynthetic and non-photosynthetic preproteins could be an adaptation that allows for the optimum balance of chloroplast proteins to be maintained at all stages of development and plastid biogenesis, as well as under conditions of biotic and abiotic stress, which can strongly influence the expression and turnover of many nucleus-encoded chloroplast proteins [82], [83], [84], [85]. It has been shown that there are ∼3000 putative precursor binding sites per chloroplast in peas [86]. Therefore, the presence of multiple receptors with overlapping preprotein recognition capabilities may also be a mechanism to ensure adequate capacity to import a range of different preproteins during early stages of development and chloroplast differentiation, when a large number of new proteins are needed to support organelle biogenesis.

Although a benefit of our yeast-based approach was the eukaryotic environment in which the protein interactions took place, there may still exist the possibility that yeast cytosol lacks an important component(s) that is involved in TP-receptor interactions in plant cells. The emerging consensus from our study is that there may exist a great degree of complexity in the mechanism of substrate selection by alternate pathways for each particular preprotein or subsets of preproteins. Similar findings were reported recently using large scale proteomic analysis and genome wide transcript profiling of Toc159 mutants which demonstrated existence of both Toc159-dependent and -independent photosynthetic and non-photosynthetic precursor proteins suggesting a non-restrictive role of this family of receptors *in vivo* [87]. A further large scale holistic approach to studying the initial recognition and binding of various precursor proteins to Toc159 receptors under various developmental stages or stress conditions could provide a greater understanding of the alternate pathways for protein targeting to chloroplasts.

## Supporting Information

Figure S1 **(A) Immunoblot analysis of whole cell extracts of NMY51 yeast strains expressing the atToc159 G-domain (Toc159G) or atToc132 G-Domain (Toc132G) baits as fusion proteins with Cub-LexA-VP16 using mouse monoclonal antibody directed against LexA.** Detection of fusion bait proteins was carried out by growing each transformant strain in SD-L medium overnight, extracting total protein and carrying out Western Blot detection as described in materials and methods. 100 µl of total protein extracts in SDS sample buffer from overnight grown strains containing Toc159G or Toc132G bait fusion proteins were loaded in lane 1 and 2, respectively. The positions of molecular markers are indicated. (B) Expression and purification of recombinant hexahistidine-tagged LHCA4(TP)-DHFR_His_, FNR1(TP)-DHFR_His_ or DHFR_His_. C-terminally His_6_-tagged versions of LHCA4(TP)-DHFR_His_, FNR1(TP)-DHFR_His_ or DHFR_His_ were expressed in *E. coli*, purified using Ni^2+^-NTA chromatography and analyzed using SDS-PAGE stained with Coomassie blue. Molecular weight markers (kDa) are indicated.
(TIF)

Figure S2 **The split-ubiquitin membrane based yeast two-hybrid analysis of the prey proteins (** **Table 1** **and** **2** **) isolated from Toc159G, Toc132G and Toc132AG screens for bait dependency.** All the prey proteins isolated (Table 1 and 2) were individually co-expressed in the *S. cerevisiae* strain NMY51 with a non-interacting negative control bait construct pTSU2-APP and re-streaked on plates with media for selecting for the presence of both bait and prey (i.e. double dropout media, SD-LW) and on plates selecting for a protein interaction (i.e. quadruple selective media supplemented with 10 mM 3-aminotriazole, SD-LWHA/3-AT) plates. Plates were incubated at 30°C for 3 days (SD-LW) and 6 days (SD-LWHA/3-AT) prior to photography. Strains co-expressing unrelated control bait protein and prey exhibit growth only on SD-LW selective media. Growth plates A and B represent the yeast colonies co-transformed with respective bait and non-photosynthetic related prey (Table 1), whereas growth plates C and D represent yeast colonies co-transformed with respective bait and photosynthetic related prey (Table 2). Names in the upper panel of each box represent the bait protein/construct and at the lower panel, represent the prey protein/construct. A positive control bait, pTSU2-APP with a positive control prey, pNubG-Fe65 (Dualsystems Biotech), were used as a positive control interaction for each set.
(TIF)

Figure S3 **Qualitative assay of the reporter gene** ***LacZ*** **using X-Gal as a substrate.** An X-Gal filter assay was carried out for all of the positive interaction colonies isolated from (A) the Toc159G-domain screen, (B) the Toc132G-domain screen, and (C) the Toc132AG screen (Table 1 and 2). Positive colonies from the screens were re-streaked on the SD-LW media plates and incubated at 30°C for 3 days prior to the assay. The test was made on filter papers as described in the material and methods. The blue coloration developed after 30 min. A positive control bait, pTSU2-APP, with a positive control prey, pNubG-Fe65 (Dualsystems Biotech), were used as a positive control interaction for each set. Colonies co-transformed with respective bait and empty library vector pR3-N were selected as negative controls. Labels in the upper panel of each box represent the bait protein/construct and those in the lower panel, represent the prey protein/construct.
(TIF)

Table S1 **Classification of non-photosynthetic (** **Table 1** **) and photosynthetic (** **Table 2** **) proteins, identified as interactors with atToc159G-, atToc132G- and atToc132AG-domain bait proteins according to biological processes.**
(DOC)
