# Supplementary figures and images for: A Split-Ubiquitin Yeast Two-Hybrid Screen to Examine the Substrate Specificity of atToc159 and atToc132, Two Arabidopsis Chloroplast Preprotein Import Receptors
Source: PLoS One. 2014 Apr 15;9(4):e95026. doi: 10.1371/journal.pone.0095026 (PMC3988174; doi:10.1371/journal.pone.0095026)

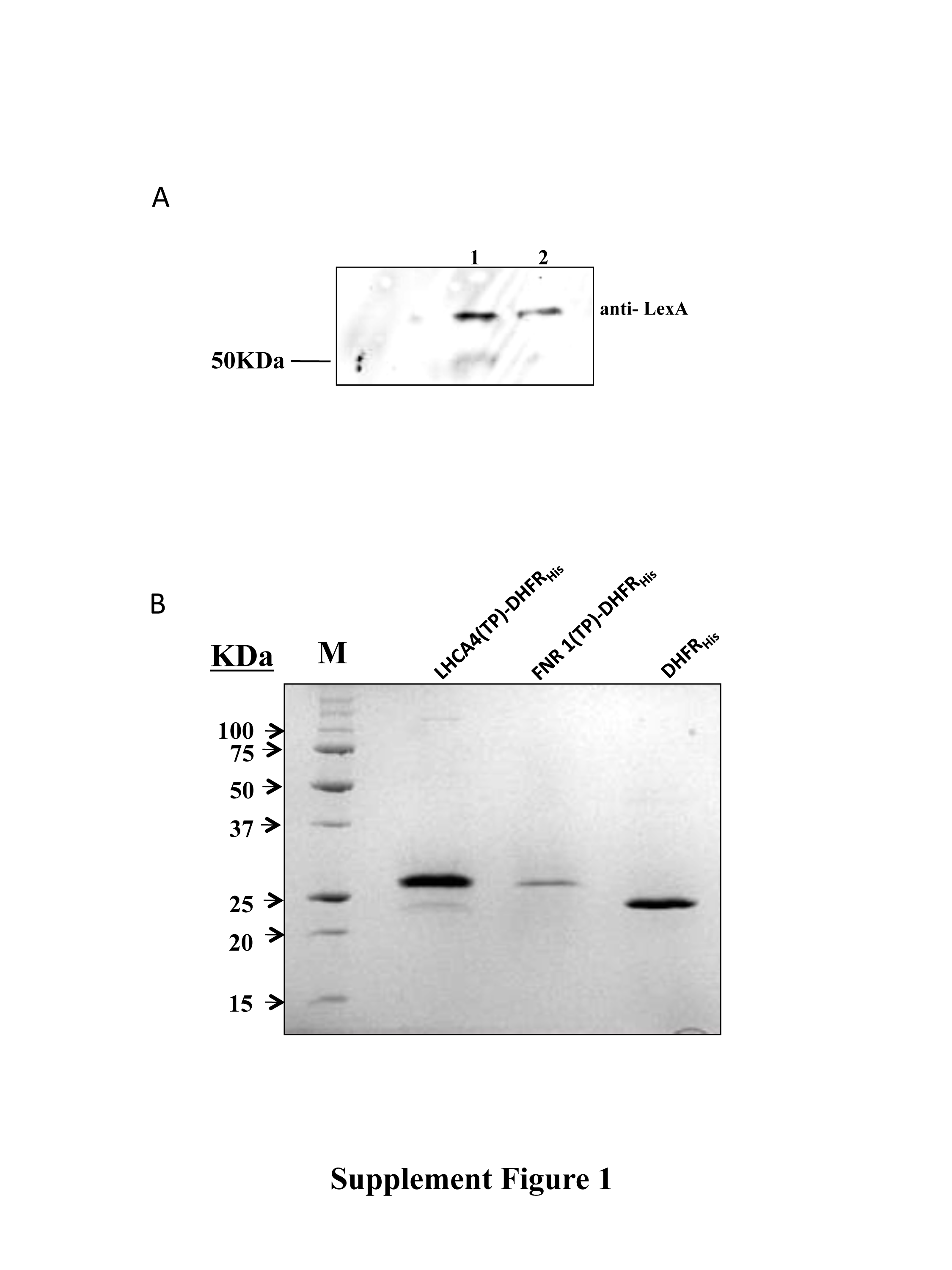

Supplement: Figure S1 — (A) Immunoblot analysis of whole cell extracts of NMY51 yeast strains expressing the atToc159 G-domain (Toc159G) or atToc132 G-Domain (Toc132G) baits as fusion proteins with Cub-LexA-VP16 using mouse monoclonal antibody directed against LexA. Detection of fusion bait proteins was carried out by growing each transformant strain in SD-L medium overnight, extracting total protein and carrying out Western Blot detection as described in materials and methods. 100 µl of total protein extracts in SDS sample buffer from overnight grown strains containing Toc159G or Toc132G bait fusion proteins were loaded in lane 1 and 2, respectively. The positions of molecular markers are indicated. (B) Expression and purification of recombinant hexahistidine-tagged LHCA4(TP)-DHFRHis, FNR1(TP)-DHFRHis or DHFRHis. C-terminally His6-tagged versions of LHCA4(TP)-DHFRHis, FNR1(TP)-DHFRHis or DHFRHis were expressed in E. coli, purified using Ni2+-NTA chromatography and analyzed using SDS-PAGE stained with Coomassie blue. Molecular weight markers (kDa) are indicated. (TIF) [file pone.0095026.s001.tif]

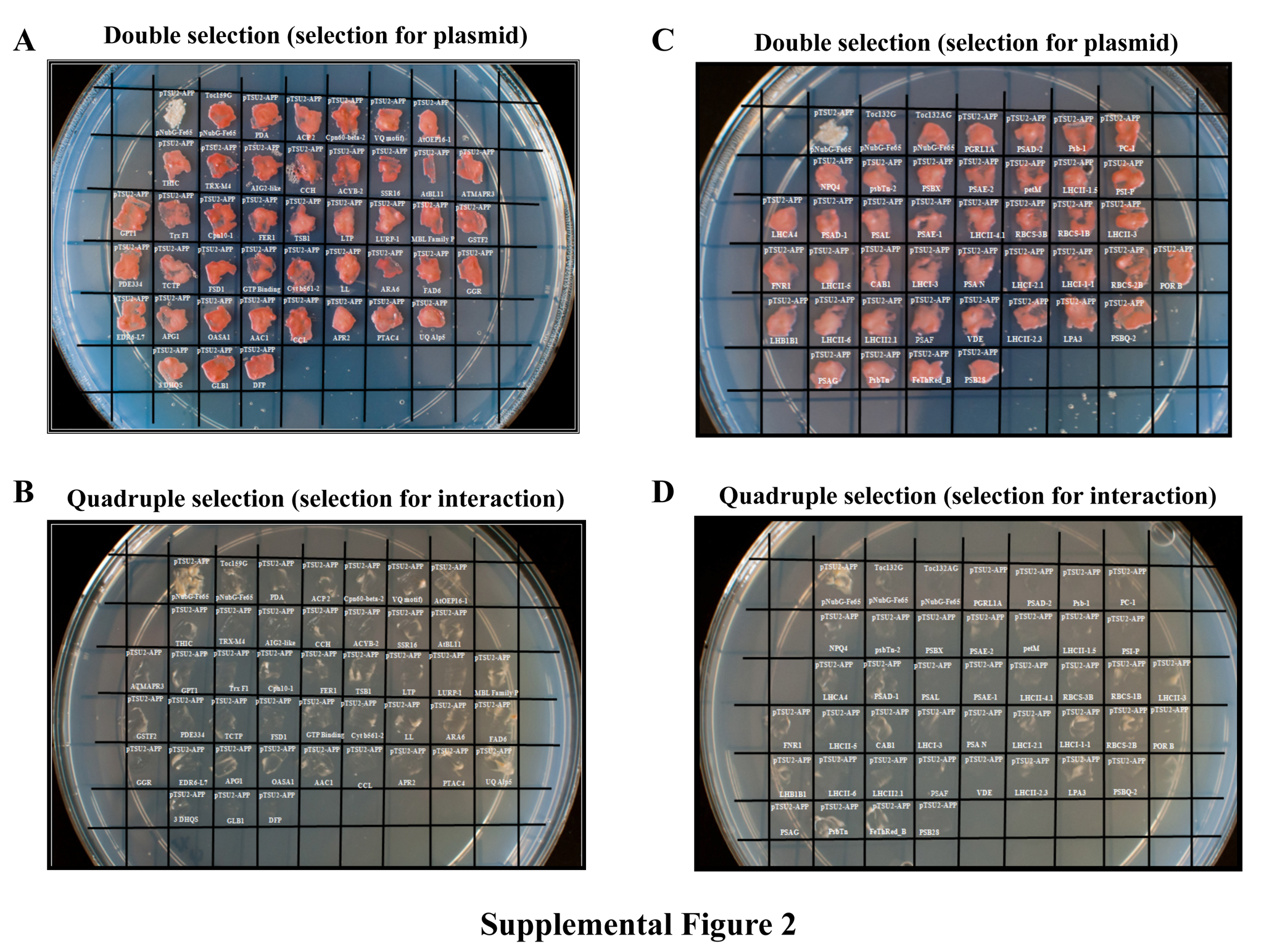

Supplement: Figure S2 — The split-ubiquitin membrane based yeast two-hybrid analysis of the prey proteins ( Table 1 and 2 ) isolated from Toc159G, Toc132G and Toc132AG screens for bait dependency. All the prey proteins isolated (Table 1 and 2) were individually co-expressed in the S. cerevisiae strain NMY51 with a non-interacting negative control bait construct pTSU2-APP and re-streaked on plates with media for selecting for the presence of both bait and prey (i.e. double dropout media, SD-LW) and on plates selecting for a protein interaction (i.e. quadruple selective media supplemented with 10 mM 3-aminotriazole, SD-LWHA/3-AT) plates. Plates were incubated at 30°C for 3 days (SD-LW) and 6 days (SD-LWHA/3-AT) prior to photography. Strains co-expressing unrelated control bait protein and prey exhibit growth only on SD-LW selective media. Growth plates A and B represent the yeast colonies co-transformed with respective bait and non-photosynthetic related prey (Table 1), whereas growth plates C and D represent yeast colonies co-transformed with respective bait and photosynthetic related prey (Table 2). Names in the upper panel of each box represent the bait protein/construct and at the lower panel, represent the prey protein/construct. A positive control bait, pTSU2-APP with a positive control prey, pNubG-Fe65 (Dualsystems Biotech), were used as a positive control interaction for each set. (TIF) [file pone.0095026.s002.tif]

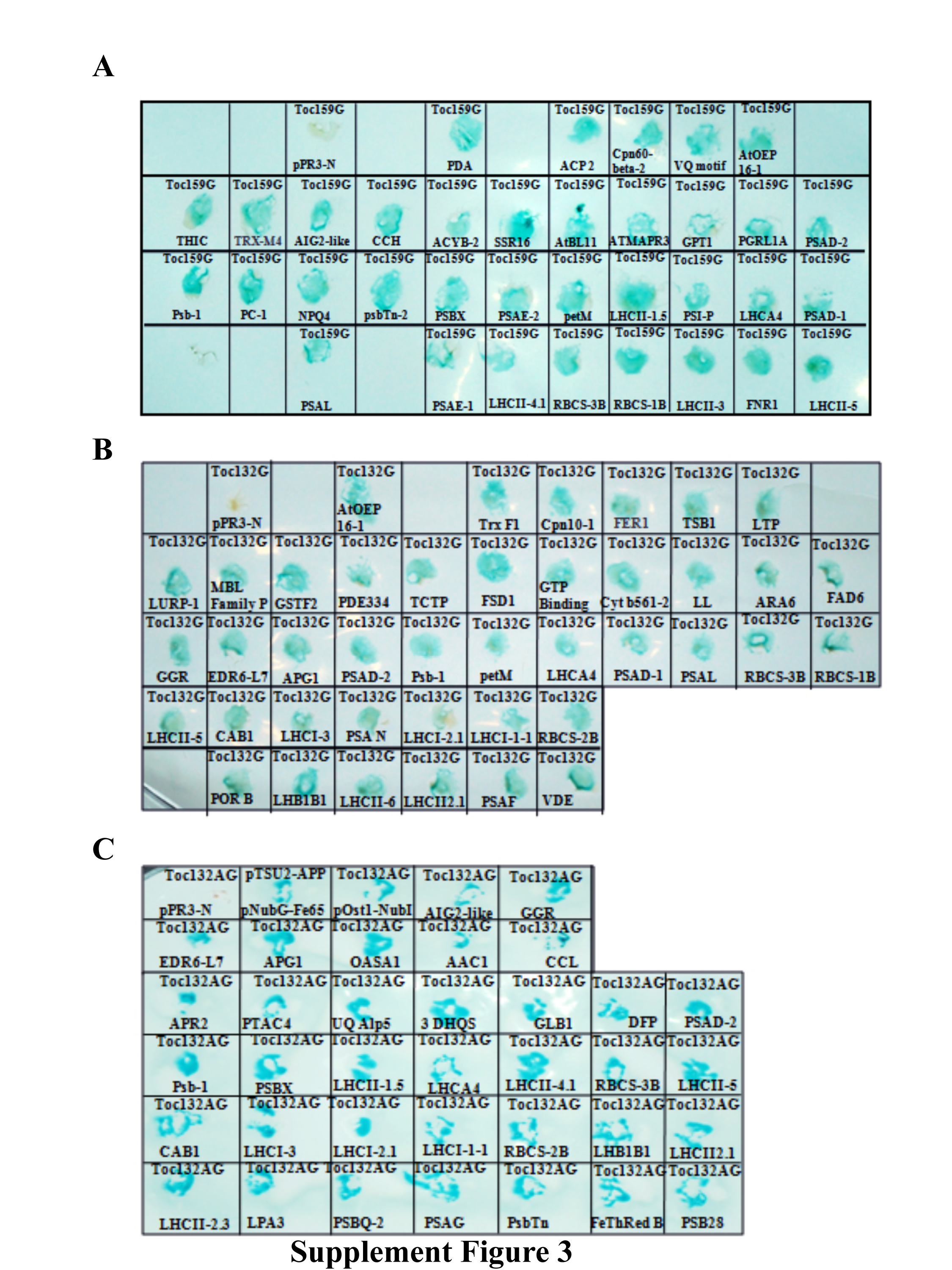

Supplement: Figure S3 — Qualitative assay of the reporter gene LacZ using X-Gal as a substrate. An X-Gal filter assay was carried out for all of the positive interaction colonies isolated from (A) the Toc159G-domain screen, (B) the Toc132G-domain screen, and (C) the Toc132AG screen (Table 1 and 2). Positive colonies from the screens were re-streaked on the SD-LW media plates and incubated at 30°C for 3 days prior to the assay. The test was made on filter papers as described in the material and methods. The blue coloration developed after 30 min. A positive control bait, pTSU2-APP, with a positive control prey, pNubG-Fe65 (Dualsystems Biotech), were used as a positive control interaction for each set. Colonies co-transformed with respective bait and empty library vector pR3-N were selected as negative controls. Labels in the upper panel of each box represent the bait protein/construct and those in the lower panel, represent the prey protein/construct. (TIF) [file pone.0095026.s003.tif]
