# Supplementary material for: A Split-Ubiquitin Yeast Two-Hybrid Screen to Examine the Substrate Specificity of atToc159 and atToc132, Two Arabidopsis Chloroplast Preprotein Import Receptors
Source: PLoS One. 2014 Apr 15;9(4):e95026. doi: 10.1371/journal.pone.0095026 (PMC3988174; doi:10.1371/journal.pone.0095026)
Supplement: Table S1 — Classification of non-photosynthetic ( Table 1 ) and photosynthetic ( Table 2 ) proteins, identified as interactors with atToc159G-, atToc132G- and atToc132AG-domain bait proteins according to biological processes. (DOC) [file pone.0095026.s004.doc]

**Table 1**. Classification of non-photosynthetic proteins, identified as interactors with atToc159G-, atToc132G- and atToc132AG-domain bait proteins according to biological processes.

| **Name of Gene** | **Biological Process** | **References** |
| --- | --- | --- |
| Plastidial Dihydrolipoamide Acetyltransferase (PDA) | Early embryo development | [1] |
| Acyl Carrier Protein 2 (ACP 2) | Fatty acid biosynthesis | [2] |
| Chaperonin 60 Beta (Cpn60-beta-2) | Molecular chaperon | [3] |
| VQ motif-containing protein (VQ motif) | UV-B stress tolerance | [4] |
| Outer Plastid Envelope Protein 16-1 (AtOEP16-1) | Seed development | [5] |
| ThiaminC (THIC) | Thiamine biosynthesis | [6] |
| Thioredoxin M-Type 4 (TRX-M4) | Redox regulator | [7] |
| AIG2-like (avirulence induced gene) family protein ( AIG2-like) | Uncharacterised | [8] |
| Copper Chaperone (CCH) | Uncharacterised | [9] |
| Cytochrome b561-2 ( ACYB2, Cyt b561-2) | Uncharacterised | [9] |
| Small Subunit Ribosomal Protein 16 (SSR16) | Ribosome biogenesis | [10] |
| Rhomboid-Like Protein 11 (AtBL11) | Jasmonic acid biosynthesis | [11] |
| Membrane-associated progesterone binding protein 3 (ATMAPR3) | Heme-binding protein | [12] |
| Glucose-6-phosphate transmembrane transporter (GPT1) | Early embryo development | [13] |
| Thioredoxin F-type 1 (Trx F1) | Carbohydrate metabolism | [14] |
| Chloroplast chaperonin 10 (Cpn10-2) | Protein folding | [15] |
| Ferretin 1 (FER1) | Stress response | [16] |
| Tryptophan synthase, beta subunit 1 (TSB1) | Tryptophan biosynthesis | [17] |
| Lipid-transfer protein (LTP) | Transport proteins | [12] |
| Protein LURP-one-related 15 Protein (LURP-1) | Uncharacterised | [8] |
| Metallo-beta-lactamase family protein (MBL Family P) | Catalytic activity | [18] |
| Glutathione S-transferase PHI 2 (GSTF2) | Stress response | [19] |
| ATPase, F0 complex, subunit B/B' (CFO-II – atpG, PDE334) | Transport proteins | [12] |
| Translationally controlled tumor protein (TCTP) | Cell growth regulation | [9] |
| Fe superoxide dismutase 1 (FSD1) | Stress response | [9] |
| Small GTP-binding protein (GTP Binding) | Translation processes | [9] |
| Lactoylglutathione lyase (LL) | Carbohydrate metabolism | [20] |
| THI1 -involved in thiamine synthesis (vitamine B) (ARA6) | Thiamine synthesis | [21] |
| Fatty acid desaturase 6 (FAD6) | Fatty acid biosynthesis | [22] |
| Geranylgeranyl reductase (GGR) | Isoprenoid biosynthesis | [9] |
| sugar transporter EDR6-like 7 (EDR6-L7) | Transport protein | [8] |
| MPBQ/MSBQ methyl transferase (APG1, E37) | Tocopherol biosynthesis | [23] |
| Cysteine synthase (OASA1) | Cysteine biosynthesis | [24] |
| ATP carrier protein 1 (AAC1) | Transmembrane transporter | [6] |
| CCR-like protein (CCL) | Circadian clock regulation | [25] |
| 5'-Adenylylsulfate reductase 2 (APR2) | Sulphate assimilation | [26] |
| Plastid transcriptionally active 4 (PTAC4) | Membrane biogenesis | [27] |
| NADH dehydrogenase (ubiquinone) 1 alpha subcomplex 5(UQ Alp5) | Oxidation-reduction function | [8] |
| Putative 3-dehydroquinate synthase (3 DHQS) | Aromatic amino acid family biosynthesis | [9] |
| GLNB1-like protein (GLB1) | Cell signalling | [28] |
| Dehydrin family protein (DFP) | Stress response | [8] |

**Table 2**. Classification of photosynthetic proteins, identified as interactors with atToc159G-, atToc132G- and atToc132AG-domain bait proteins according to biological processes.

| **Name of Gene** | **Biological Processes** | **References** |
| --- | --- | --- |
| PGR5-Like A (PGRL1A) | Electron transfer components | [29] |
| Photosystem I subunit D-2 (PSAD-2) | Photosystem I associated protein | [30] |
| Photosystem II Subunit P-1 (Psb-1) | Photosystem II associated protein | [31] |
| Plastocyanin 2 (PETE2) | Electron transfer components | [32] |
| Photosystem II Subunit S (NPQ4, psdS) | Photosystem II associated protein | [33] |
| Photosystem II 5 kD protein (psbTn-2) | Photosystem II associated protein | [34] |
| Photosystem II subunit X (PSBX) | Photosystem II associated protein | [35] |
| Photosystem I subunit E-2 (PSAE-2) | Photosystem I associated protein | [36] |
| Cytochrome b6f complex subunit M (petM) | Electron transfer components | [12] |
| Photosystem II light harvesting complex gene B1B2 (LHCII-1.5) | Photosystem II associated protein | [37] |
| Photosystem I P Subunit (PSI-P) | Photosystem I associated protein | [31] |
| Light-Harvesting Chloroplyll-Protein Complex I Subunit A4 (LHCA4) | Photosystem I associated protein | [38] |
| Photosystem I subunit D-1( PSAD-1) | Photosystem I associated protein | [30] |
| Photosystem I subunit L (PSAL) | Photosystem I associated protein | [39] |
| Photosystem I reaction centre subunit IV (PSAE-1) | Photosystem I associated protein | [40] |
| Light harvesting complex photosystem II (LHCII-4.1) | Photosystem II associated protein | [41] |
| RuBisCO small subunit 3B (RBCS-3B) | Carbon fixation | [42] |
| RuBisCO small subunit 1B (RBCS-1B) | Carbon fixation | [42] |
| Light-Harvesting Chloroplyll B-Binding Protein 3 (LHCII-3) | Photosystem II associated protein | [43] |
| Ferredoxin-NADP(+)-oxidoreductase 1 (FNR1) | Electron transfer components | [44] |
| Light Harvesting Complex Of Photosystem II 5 (LHCII-5) | Photosystem II associated protein | [45] |
| Chlorophyll A/B binding protein 1 (CAB1) | Photosystem II associated protein | [46] |
| Photosystem I light harvesting complex gene 3 (LHCI-3) | Photosystem I associated protein | [47] |
| Photosystem I reaction center subunit PSI-N (PSAN) | Photosystem I associated protein | [48] |
| Photosystem I light harvesting complex gene 2 (LHCI-2.1) | Photosystem I associated protein | [49] |
| Photosystem I light harvesting complex gene 1 (LHCI-1-1) | Photosystem I associated protein | [50] |
| Rubisco small subunit 2b (RBCS-2B) | Carbon fixation | [42] |
| Protochlorophyllide oxidoreductase B (POR B) | Chlorophyll biosynthesis | [51] |
| Light-harvesting chlorophyll-protein complex II subunit B1 (LHB1B1) |  | [52] |
| Light harvesting complex photosystem II subunit 6 (LHCII-6) | Photosystem II associated protein | [53] |
| Photosystem II light harvesting complex gene 2.1 (LHCII2.1) | Photosystem II associated protein | [54] |
| Photosystem I subunit F (PSAF) | Photosystem I associated protein | [55] |
| Violaxanthin Deepoxidase (VDE, NPQ1) | Xanthophyll cycle | [56] |
| Photosystem II light harvesting complex protein 2.3 (LHCII-2.3) | Photosystem II associated protein | [54] |
| Low PSII accumulation 3 protein (LPA3) | Photosystem II associated protein | [57] |
| Oxygen-evolving enhancer protein 3-2 (PSBQ-2) | Photosystem II associated protein | [58] |
| Photosystem I subunit G (PSAG) | Photosystem I associated protein | [59] |
| Photosystem II subunit T (PsbTn) | Photosystem II associated protein | [60] |
| Ferredoxin-thioredoxin reductase subunit A (FeThRed_B) | Photosynthetic enzyme regulator | [61] |
| Photosystem II reaction center PSB28 protein (PSB28) | Photosystem II associated protein | [62] |

1. Lin M, Behal R, Oliver DJ (2003) Disruption of plE2, the gene for the E2 subunit of the plastid pyruvate dehydrogenase complex, in Arabidopsis causes an early embryo lethal phenotype. Plant Mol Biol 52: 865-872.

2. Lamppa G, Jacks C (1991) Analysis of two linked genes coding for the acyl carrier protein (ACP) from Arabidopsis thaliana (columbia). Plant Mol Biol 16: 469-474.

3. Suzuki K, Nakanishi H, Bower J, Yoder DW, Osteryoung KW, et al. (2009) Plastid chaperonin proteins Cpn60 alpha and Cpn60 beta are required for plastid division in Arabidopsis thaliana. BMC Plant Biol 9: 38.

4. Gonzalez Besteiro MA, Bartels S, Albert A, Ulm R Arabidopsis MAP kinase phosphatase 1 and its target MAP kinases 3 and 6 antagonistically determine UV-B stress tolerance, independent of the UVR8 photoreceptor pathway. Plant J 68: 727-737.

5. Samol I, Rossig C, Buhr F, Springer A, Pollmann S, et al. The outer chloroplast envelope protein OEP16-1 for plastid import of NADPH:protochlorophyllide oxidoreductase A in Arabidopsis thaliana. Plant Cell Physiol 52: 96-111.

6. Kong D, Zhu Y, Wu H, Cheng X, Liang H, et al. (2008) AtTHIC, a gene involved in thiamine biosynthesis in Arabidopsis thaliana. Cell Res 18: 566-576.

7. Gelhaye E, Rouhier N, Navrot N, Jacquot JP (2005) The plant thioredoxin system. Cell Mol Life Sci 62: 24-35.

8. Kleffmann T, Russenberger D, von Zychlinski A, Christopher W, Sjolander K, et al. (2004) The Arabidopsis thaliana chloroplast proteome reveals pathway abundance and novel protein functions. Curr Biol 14: 354-362.

9. Zybailov B, Rutschow H, Friso G, Rudella A, Emanuelsson O, et al. (2008) Sorting signals, N-terminal modifications and abundance of the chloroplast proteome. PLoS One 3: e1994.

10. Ueda M, Nishikawa T, Fujimoto M, Takanashi H, Arimura S, et al. (2008) Substitution of the gene for chloroplast RPS16 was assisted by generation of a dual targeting signal. Mol Biol Evol 25: 1566-1575.

11. Knopf RR, Feder A, Mayer K, Lin A, Rozenberg M, et al. Rhomboid proteins in the chloroplast envelope affect the level of allene oxide synthase in Arabidopsis thaliana. Plant J 72: 559-571.

12. Peltier JB, Ytterberg AJ, Sun Q, van Wijk KJ (2004) New functions of the thylakoid membrane proteome of Arabidopsis thaliana revealed by a simple, fast, and versatile fractionation strategy. J Biol Chem 279: 49367-49383.

13. Andriotis VM, Pike MJ, Bunnewell S, Hills MJ, Smith AM The plastidial glucose-6-phosphate/phosphate antiporter GPT1 is essential for morphogenesis in Arabidopsis embryos. Plant J 64: 128-139.

14. Thormahlen I, Ruber J, von Roepenack-Lahaye E, Ehrlich SM, Massot V, et al. Inactivation of thioredoxin f1 leads to decreased light activation of ADP-glucose pyrophosphorylase and altered diurnal starch turnover in leaves of Arabidopsis plants. Plant Cell Environ 36: 16-29.

15. Vitlin A, Weiss C, Demishtein-Zohary K, Rasouly A, Levin D, et al. Chloroplast beta chaperonins from A. thaliana function with endogenous cpn10 homologs in vitro. Plant Mol Biol 77: 105-115.

16. Touraine B, Briat JF, Gaymard F GSH threshold requirement for NO-mediated expression of the Arabidopsis AtFer1 ferritin gene in response to iron. FEBS Lett 586: 880-883.

17. Jing Y, Cui D, Bao F, Hu Z, Qin Z, et al. (2009) Tryptophan deficiency affects organ growth by retarding cell expansion in Arabidopsis. Plant J 57: 511-521.

18. Catarecha P, Segura MD, Franco-Zorrilla JM, Garcia-Ponce B, Lanza M, et al. (2007) A mutant of the Arabidopsis phosphate transporter PHT1;1 displays enhanced arsenic accumulation. Plant Cell 19: 1123-1133.

19. Dixon DP, Hawkins T, Hussey PJ, Edwards R (2009) Enzyme activities and subcellular localization of members of the Arabidopsis glutathione transferase superfamily. J Exp Bot 60: 1207-1218.

20. Mustafiz A, Singh AK, Pareek A, Sopory SK, Singla-Pareek SL Genome-wide analysis of rice and Arabidopsis identifies two glyoxalase genes that are highly expressed in abiotic stresses. Funct Integr Genomics 11: 293-305.

21. Chabregas SM, Luche DD, Farias LP, Ribeiro AF, van Sluys MA, et al. (2001) Dual targeting properties of the N-terminal signal sequence of Arabidopsis thaliana THI1 protein to mitochondria and chloroplasts. Plant Mol Biol 46: 639-650.

22. Fan J, Xu C Genetic analysis of Arabidopsis mutants impaired in plastid lipid import reveals a role of membrane lipids in chloroplast division. Plant Signal Behav 6: 458-460.

23. Motohashi R, Ito T, Kobayashi M, Taji T, Nagata N, et al. (2003) Functional analysis of the 37 kDa inner envelope membrane polypeptide in chloroplast biogenesis using a Ds-tagged Arabidopsis pale-green mutant. Plant J 34: 719-731.

24. Olinares PD, Ponnala L, van Wijk KJ Megadalton complexes in the chloroplast stroma of Arabidopsis thaliana characterized by size exclusion chromatography, mass spectrometry, and hierarchical clustering. Mol Cell Proteomics 9: 1594-1615.

25. Lidder P, Gutierrez RA, Salome PA, McClung CR, Green PJ (2005) Circadian control of messenger RNA stability. Association with a sequence-specific messenger RNA decay pathway. Plant Physiol 138: 2374-2385.

26. Mugford SG, Lee BR, Koprivova A, Matthewman C, Kopriva S Control of sulfur partitioning between primary and secondary metabolism. Plant J 65: 96-105.

27. Kroll D, Meierhoff K, Bechtold N, Kinoshita M, Westphal S, et al. (2001) VIPP1, a nuclear gene of Arabidopsis thaliana essential for thylakoid membrane formation. Proc Natl Acad Sci U S A 98: 4238-4242.

28. Chen YM, Ferrar TS, Lohmeier-Vogel EM, Morrice N, Mizuno Y, et al. (2006) The PII signal transduction protein of Arabidopsis thaliana forms an arginine-regulated complex with plastid N-acetyl glutamate kinase. J Biol Chem 281: 5726-5733.

29. DalCorso G, Pesaresi P, Masiero S, Aseeva E, Schunemann D, et al. (2008) A complex containing PGRL1 and PGR5 is involved in the switch between linear and cyclic electron flow in Arabidopsis. Cell 132: 273-285.

30. Ihnatowicz A, Pesaresi P, Varotto C, Richly E, Schneider A, et al. (2004) Mutants for photosystem I subunit D of Arabidopsis thaliana: effects on photosynthesis, photosystem I stability and expression of nuclear genes for chloroplast functions. Plant J 37: 839-852.

31. Khrouchtchova A, Hansson M, Paakkarinen V, Vainonen JP, Zhang S, et al. (2005) A previously found thylakoid membrane protein of 14kDa (TMP14) is a novel subunit of plant photosystem I and is designated PSI-P. FEBS Lett 579: 4808-4812.

32. Pesaresi P, Scharfenberg M, Weigel M, Granlund I, Schroder WP, et al. (2009) Mutants, overexpressors, and interactors of Arabidopsis plastocyanin isoforms: revised roles of plastocyanin in photosynthetic electron flow and thylakoid redox state. Mol Plant 2: 236-248.

33. Goral TK, Johnson MP, Duffy CD, Brain AP, Ruban AV, et al. Light-harvesting antenna composition controls the macrostructure and dynamics of thylakoid membranes in Arabidopsis. Plant J 69: 289-301.

34. Friso G, Giacomelli L, Ytterberg AJ, Peltier JB, Rudella A, et al. (2004) In-depth analysis of the thylakoid membrane proteome of Arabidopsis thaliana chloroplasts: new proteins, new functions, and a plastid proteome database. Plant Cell 16: 478-499.

35. Garcia-Cerdan JG, Sveshnikov D, Dewez D, Jansson S, Funk C, et al. (2009) Antisense inhibition of the PsbX protein affects PSII integrity in the higher plant Arabidopsis thaliana. Plant Cell Physiol 50: 191-202.

36. Ihnatowicz A, Pesaresi P, Leister D (2007) The E subunit of photosystem I is not essential for linear electron flow and photoautotrophic growth in Arabidopsis thaliana. Planta 226: 889-895.

37. Pesaresi P, Masiero S, Eubel H, Braun HP, Bhushan S, et al. (2006) Nuclear photosynthetic gene expression is synergistically modulated by rates of protein synthesis in chloroplasts and mitochondria. Plant Cell 18: 970-991.

38. Ihnatowicz A, Pesaresi P, Lohrig K, Wolters D, Muller B, et al. (2008) Impaired photosystem I oxidation induces STN7-dependent phosphorylation of the light-harvesting complex I protein Lhca4 in Arabidopsis thaliana. Planta 227: 717-722.

39. An D, Yang J, Zhang P Transcriptome profiling of low temperature-treated cassava apical shoots showed dynamic responses of tropical plant to cold stress. BMC Genomics 13: 64.

40. Lehtimaki N, Lintala M, Allahverdiyeva Y, Aro EM, Mulo P Drought stress-induced upregulation of components involved in ferredoxin-dependent cyclic electron transfer. J Plant Physiol 167: 1018-1022.

41. de Bianchi S, Betterle N, Kouril R, Cazzaniga S, Boekema E, et al. Arabidopsis mutants deleted in the light-harvesting protein Lhcb4 have a disrupted photosystem II macrostructure and are defective in photoprotection. Plant Cell 23: 2659-2679.

42. Izumi M, Tsunoda H, Suzuki Y, Makino A, Ishida H RBCS1A and RBCS3B, two major members within the Arabidopsis RBCS multigene family, function to yield sufficient Rubisco content for leaf photosynthetic capacity. J Exp Bot 63: 2159-2170.

43. Kouril R, Wientjes E, Bultema JB, Croce R, Boekema EJ High-light vs. low-light: effect of light acclimation on photosystem II composition and organization in Arabidopsis thaliana. Biochim Biophys Acta 1827: 411-419.

44. Lintala M, Lehtimaki N, Benz JP, Jungfer A, Soll J, et al. Depletion of leaf-type ferredoxin-NADP(+) oxidoreductase results in the permanent induction of photoprotective mechanisms in Arabidopsis chloroplasts. Plant J 70: 809-817.

45. Nick S, Meurer J, Soll J, Ankele E Nucleus-encoded light-harvesting chlorophyll a/b proteins are imported normally into chlorophyll b-free chloroplasts of Arabidopsis. Mol Plant 6: 860-871.

46. Yakushevska AE, Jensen PE, Keegstra W, van Roon H, Scheller HV, et al. (2001) Supermolecular organization of photosystem II and its associated light-harvesting antenna in Arabidopsis thaliana. Eur J Biochem 268: 6020-6028.

47. Mozzo M, Morosinotto T, Bassi R, Croce R (2006) Probing the structure of Lhca3 by mutation analysis. Biochim Biophys Acta 1757: 1607-1613.

48. Stael S, Rocha AG, Wimberger T, Anrather D, Vothknecht UC, et al. Cross-talk between calcium signalling and protein phosphorylation at the thylakoid. J Exp Bot 63: 1725-1733.

49. Ganeteg U, Strand A, Gustafsson P, Jansson S (2001) The properties of the chlorophyll a/b-binding proteins Lhca2 and Lhca3 studied in vivo using antisense inhibition. Plant Physiol 127: 150-158.

50. Wientjes E, van Stokkum IH, van Amerongen H, Croce R Excitation-energy transfer dynamics of higher plant photosystem I light-harvesting complexes. Biophys J 100: 1372-1380.

51. Frick G, Su Q, Apel K, Armstrong GA (2003) An Arabidopsis porB porC double mutant lacking light-dependent NADPH:protochlorophyllide oxidoreductases B and C is highly chlorophyll-deficient and developmentally arrested. Plant J 35: 141-153.

52. Dong H, Deng Y, Mu J, Lu Q, Wang Y, et al. (2007) The Arabidopsis Spontaneous Cell Death1 gene, encoding a zeta-carotene desaturase essential for carotenoid biosynthesis, is involved in chloroplast development, photoprotection and retrograde signalling. Cell Res 17: 458-470.

53. Marin A, Passarini F, van Stokkum IH, van Grondelle R, Croce R Minor complexes at work: light-harvesting by carotenoids in the photosystem II antenna complexes CP24 and CP26. Biophys J 100: 2829-2838.

54. Leoni C, Pietrzykowska M, Kiss AZ, Suorsa M, Ceci LR, et al. Very rapid phosphorylation kinetics suggest a unique role for Lhcb2 during state transitions in Arabidopsis. Plant J.

55. Haldrup A, Simpson DJ, Scheller HV (2000) Down-regulation of the PSI-F subunit of photosystem I (PSI) in Arabidopsis thaliana. The PSI-F subunit is essential for photoautotrophic growth and contributes to antenna function. J Biol Chem 275: 31211-31218.

56. Chen Z, Gallie DR Violaxanthin de-epoxidase is rate-limiting for non-photochemical quenching under subsaturating light or during chilling in Arabidopsis. Plant Physiol Biochem 58: 66-82.

57. Cai W, Ma J, Chi W, Zou M, Guo J, et al. Cooperation of LPA3 and LPA2 is essential for photosystem II assembly in Arabidopsis. Plant Physiol 154: 109-120.

58. Granlund I, Hall M, Kieselbach T, Schroder WP (2009) Light induced changes in protein expression and uniform regulation of transcription in the thylakoid lumen of Arabidopsis thaliana. PLoS One 4: e5649.

59. Varotto C, Pesaresi P, Jahns P, Lessnick A, Tizzano M, et al. (2002) Single and double knockouts of the genes for photosystem I subunits G, K, and H of Arabidopsis. Effects on photosystem I composition, photosynthetic electron flow, and state transitions. Plant Physiol 129: 616-624.

60. Sun Q, Emanuelsson O, van Wijk KJ (2004) Analysis of curated and predicted plastid subproteomes of Arabidopsis. Subcellular compartmentalization leads to distinctive proteome properties. Plant Physiol 135: 723-734.

61. Dal Bosco C, Lezhneva L, Biehl A, Leister D, Strotmann H, et al. (2004) Inactivation of the chloroplast ATP synthase gamma subunit results in high non-photochemical fluorescence quenching and altered nuclear gene expression in Arabidopsis thaliana. J Biol Chem 279: 1060-1069.

62. Zhang X, Wollenweber B, Jiang D, Liu F, Zhao J (2008) Water deficits and heat shock effects on photosynthesis of a transgenic Arabidopsis thaliana constitutively expressing ABP9, a bZIP transcription factor. J Exp Bot 59: 839-848.
